# Validation of a Harmonized Enzyme-Linked-Lectin-Assay (ELLA-NI) Based Neuraminidase Inhibition Assay Standard Operating Procedure (SOP) for Quantification of N1 Influenza Antibodies and the Use of a Calibrator to Improve the Reproducibility of the ELLA-NI With Reverse Genetics Viral and Recombinant Neuraminidase Antigens: A FLUCOP Collaborative Study

**DOI:** 10.3389/fimmu.2022.909297

**Published:** 2022-06-17

**Authors:** Marie-Clotilde Bernard, Joanna Waldock, Sylvie Commandeur, Lea Strauß, Claudia Maria Trombetta, Serena Marchi, Fan Zhou, Serge van de Witte, Peter van Amsterdam, Sammy Ho, Katja Hoschler, Vladimir Lugovtsev, Jerry P. Weir, Emanuele Montomoli, Rebecca J. Cox, Othmar G. Engelhardt, Damien Friel, Ralf Wagner, Thierry Ollinger, Sophie Germain, Hanna Sediri-Schön

**Affiliations:** ^1^ Department of Research and Development, Sanofi Pasteur, Marcy L’Etoile, France; ^2^ Influenza Resource Centre, National Institute for Biological Standards and Control, Potters Bar, United Kingdom; ^3^ Section viral vaccines, Virology Division, Paul-Ehrlich-Institut, Federal Institute for Vaccines and Biomedicines, Langen, Germany; ^4^ Department of Molecular and Developmental Medicine, University of Siena, Siena, Italy; ^5^ Influenza Centre, Department of Clinical Sciences, University of Bergen, Bergen, Norway; ^6^ Abbott Healthcare Products B.V., Weesp, Netherlands; ^7^ UK Health Security Agency, Colindale, United Kingdom; ^8^ Laboratory of DNA Viruses, Division of Viral Products, Office of Vaccines Research and Review, Center for Biologics Evaluation and Research, Food and Drug Administration, Silver Spring, MD, United States; ^9^ GlaxoSmithKline (GSK), Wavre, Belgium

**Keywords:** influenza, enzyme-linked lectin assay (ELLA), neuraminidase inhibition (NI) assay, serology, standardization

## Abstract

Current vaccination strategies against influenza focus on generating an antibody response against the viral haemagglutination surface protein, however there is increasing interest in neuraminidase (NA) as a target for vaccine development. A critical tool for development of vaccines that target NA or include an NA component is available validated serology assays for quantifying anti-NA antibodies. Additionally serology assays have a critical role in defining correlates of protection in vaccine development and licensure. Standardisation of these assays is important for consistent and accurate results. In this study we first validated a harmonized enzyme-linked lectin assay (ELLA)- Neuraminidase Inhibition (NI) SOP for N1 influenza antigen and demonstrated the assay was precise, linear, specific and robust within classical acceptance criteria for neutralization assays for vaccine testing. Secondly we tested this SOP with NA from influenza B viruses and showed the assay performed consistently with both influenza A and B antigens. Third, we demonstrated that recombinant NA (rNA) could be used as a source of antigen in ELLA-NI. In addition to validating a harmonized SOP we finally demonstrated a clear improvement in inter-laboratory agreement across several studies by using a calibrator. Importantly we showed that the use of a calibrator significantly improved agreement when using different sources of antigen in ELLA-NI, namely reverse genetics viruses and recombinant NA. We provide a freely available and detailed harmonized SOP for ELLA-NI. Our results add to the growing body of evidence in support of developing biological standards for influenza serology.

## Introduction

Haemagglutinin (HA) and neuraminidase (NA) are the two major surface glycoproteins of influenza viruses. Both recognize sialic acid (SA) playing different roles, the HA binds to SA on the host cells allowing virus entry, while the NA has enzymatic activity, removing SA and facilitating the release of progeny virus (1, 2). Currently, 18 HA and 11 NA subtypes have been identified, only a subset of which has been reported in human influenza virus infections.

Since the HA represents the primary target of the antibody response and correlates of protection have been established, vaccine immunogenicity is mainly evaluated on HA specific antibody response (3–5). However, NA inhibiting (NI) antibodies seem to have an independent role in protection, not associated with the prevention of infection, but contributing significantly to immune protection by reducing the severity and duration of infection and by curbing viral shedding and transmission (6). NI antibody titres have been shown to be an independent correlate of protection against influenza disease severity (7, 8). The great advantage of targeting NA is its slower antigenic evolution (9) and the ability to induce longer lasting immunity and cross-protection than that provided by conventional HA-based vaccines (7, 10–12). Some factors such as the immunodominance of the HA, the lack of regulated NA content in vaccine composition and of standardised assays have hindered the study of NI antibodies (5, 7, 13). In 2008, the World Health Organization highlighted the need to further study the role of NA and to develop simpler and more reproducible assays for detecting NI antibodies (14). In 2016, the European Medicines Agency updated the regulatory guidelines on influenza vaccines to include the possibility of evaluating NI antibodies (15).

Currently the most common and widely used technique to evaluate NI antibodies is the enzyme-linked lectin assay (ELLA), originally developed by Lambré et al. (12, 16). The assay is based on the ability of NA to cleave SA residues from a substrate, usually fetuin coated on the surface of 96-well plates. Removal of SA exposes a galactose residue, which is bound by a lectin [peanut agglutinin (PNA)] conjugated to horseradish peroxidase (HRP). The measured optical density (OD) is proportional to the NA activity in the tested samples. The ELLA-Neuraminidase Inhibition (ELLA-NI) titre is defined as the highest serum dilution that shows at least 50% inhibition of the NA activity (17, 18). ELLA is more practical than the traditional thiobarbituric acid (TBA) assay. The TBA assay is based on the detection of free SA, but is cumbersome in nature, uses hazardous reagents, and is not suited to high-throughput testing required for serology studies and NA antigenic characterisation during influenza surveillance (18–22). ELLA-NI and TBA NI titres have been shown to correlate well, however ELLA demonstrates higher sensitivity (23).

One crucial aspect of ELLA-NI is the source of NA since antibodies against HA can interfere and non-specifically inhibit NA activity through a proposed mechanism of steric hindrance (24). To avoid this possible interference, reverse genetics (RG) viruses with antigenically-mismatched HA subtypes, for which human serum samples have no antibodies, has been used for influenza A viruses (18, 19, 22). Other approaches have been evaluated, such as virus-like particles (25, 26), purified recombinant NA (rNA) using a baculovirus expression system (23) and detergent split wild-type viruses (27). Some of these approaches could be useful alternative sources of NA where mismatched RG viruses are not available (19, 20, 28).

To date ELLA-NI has been assessed in an intra-laboratory study (19) evaluating the reproducibility of the ELLA-NI. This study showed that plate-to-plate variability was minimal, the same plate was highly reproducible, and the assay was subtype specific. A subsequent inter-laboratory study (21) confirmed the assay reproducibility even across different laboratories and highlighted the importance of inclusion of a serum standard for the normalization of the NI antibody titres and reduction of variability in results. In addition, the study showed that the antigen titration is a crucial step before performing ELLA-NI, and an amount of antigen within the linear range of the titration curve should be used. Currently the ELLA-NI has been used for evaluating NI titres in several clinical influenza vaccine studies (23, 27, 29).

In this study we build upon previous work, developed and validated a consensus SOP in an international study involving 7 FLUCOP partners. FLUCOP (http://www.flucop.eu/) is a joint European project between academia, vaccine manufacturers and public health authorities, supported by the Innovative Medicines Initiative Joint Undertaking (IMIJU) aimed at standardising serological assays and developing common protocols for evaluating influenza vaccines. The goal of the FLUCOP project is to have a direct and evidence-based impact on the quality of regulatory decisions and to provide valid and appropriate serological tools for the future definition of alternative correlates of protection for (novel) influenza vaccines. In this study we present a freely available and detailed SOP for testing serum samples using ELLA-NI. We demonstrated this assay was precise, linear, robust within defined limits across multiple testing laboratories, and had subtype specificity. We show this SOP could be used to test both A, and for the first time B (both Yamagata and Victoria lineage), influenza RG viruses. Additionally we demonstrated that rNA could be used as a source of antigen in the assay, with highly reproducible results between laboratories and antigen sources when a calibrator was used to normalise results.

## Materials and Methods

### Antigens and Recombinant Proteins

RG influenza viruses used in this study are described in the **Table 1**. All viruses were propagated in chicken eggs. B viruses were inactivated using β-propriolactone. For H1N1 NA containing viruses, a combination of H7 and H9 RG viruses were used due to differences in BSL of these antigens within different countries. For this study all viral antigens used were BSL2. Recombinant proteins used as antigen or in competition assays are also listed in **Table 1**. Recombinant Na (rNA) proteins were produced in Chinese Hamster Ovaries (CHO) cells

**Table 1:** Reverse Genetics (RG) viruses and recombinant proteins use in the study.

Antigen	HA strain	NA strain
***RG viruses***		
A/H7N1	A/Equine/Prague/56 (H7N7)	A/California/07/2009 (H1N1)
A/H9N1	A/chicken/Beijing/2/97 (H9N2)	A/California/07/2009 (H1N1)
H9-NB/Brisbane	A/chicken/Beijing/2/97 (H9N2)	B/Brisbane/60/2008 (Victoria lineage)
H9-NB/Phuket	A/chicken/Beijing/2/97 (H9N2)	B/Phuket/3073/2013 (Yamagata lineage)
***Recombinant proteins***		
rHA	A/California/07/2009 (H1N1) influenza (Protein Sciences)	
gB CMV (Sanofi Pasteur)		
rNS1 (JEV, The Native Antigen Company)		
rNA (N1) Tetrabrachion folder		A/Belgium/145-MA/2009 (H1N1) (ThermoFisher Scientific)
rNA (N2) Tetrabrachion folder		A/Hong Kong/4801/2014 (H3N2) (ThermoFisher Scientific)
rNA (B Victoria) Tetrabrachion folder		B/Brisbane/60/2008 (Victoria) (ThermoFisher Scientific)
rNA (B Yamagata) Tetrabrachion folder		B/Phuket/3073/2013 (Yamagata) (ThermoFisher Scientific)

### Generation of Influenza B RG Viruses

The chimeric viruses containing HA of H9 and NAs from influenza B viruses were generated by reverse genetics technique using the pHW2000 plasmid as described earlier (30). The HA of these strains is a chimeric protein consisting of the HA ectodomain from H9N2 strain A/Chicken/Beijing/2/97 (H9N2), and the CT+TM (cytoplasmic tail + transmembrane region) from seasonal H1N1 strain A/Brisbane/59/2007. The NA of these viruses are also chimeric proteins containing an ectodomain (stalk and head) of the influenza B viruses (B/Brisbane/60/2008 or B/Phuket/3073/2013) and CT+TM from seasonal H1N1 strain A/Brisbane/59/2007 (**Supplementary Figure S1**).

### Clinical Serum Samples

For the end of run study, participating laboratories were asked to select their own panel of 6 in-house human serum samples for testing. For Precision studies a panel of 9 post-vaccination human serum samples and for Linearity a panel of 4 post-vaccination human serum samples were provided to each participating laboratory by Sanofi Pasteur (2015-2016 trivalent influenza vaccine (TIV) (A/California/07/2009, A/South Australia/55/2014, B/Phuket/3073/2013) or 2015-2016 quadrivalent influenza vaccine (QIV) (A/California/07/2009, A/South Australia/55/2014, B/Phuket/3073/2013, B/Brisbane/60/2008)). For testing Robustness and testing B viruses/rNA, a panel of 12 and 16 pre and post-vaccination human serum samples respectively were provided to each participating laboratory by the University of Ghent (Flucop_QIV clinical trial, Fluarix Tetra vaccine containing the following influenza strains: A/Michigan/45/2015 (H1N1)pdm09, A/Hong Kong/4801/2014 (H3N2), B/Brisbane/60/2008 and B/Phuket/3073/2013). Prior to the studies, serum samples were pre-screened in ELLA-NI and selected to cover the dynamic range of the assay. All sera were heat inactivated at 56°C for 1 hour. Serum minus IgA/IgM/IgG (human) was used as a negative control (Sigma-Aldrich S5393).

For the HA competition analysis, 9w-old Female BALB/cByJ mice (Charles River - 327 impasse du domaine Rozier, 69210 Saint-Germain-Nuelles, France) were immunized twice at D0 and D28 and blood samples collected at D49 were pooled. Three mouse sera were tested: a pool of sera from mice vaccinated with monovalent H1N1pdm09 vaccine (monovalent A/California/07/2009, Sanofi Pasteur; sera positive for H1 and N1 antibodies, Haemagglutination Inhibition assay (HAI) titre 160); a serum from a mouse inoculated with rHA (A/California/07/2009) (positive for H1 antibodies, HAI titre 640) and a pool of sera from mice inoculated with PBS (negative for H1 and N1 antibodies).

For specificity, monospecific sera from six individual ferrets infected with wild type (WT) influenza viruses (2 ferrets with A/California/07/2009, 2 ferrets with B/Brisbane/60/2008 and 2 ferrets with B/Phuket/3073/2013) were tested (4 ferrets from Highgate Farm, Male, ages 6 months, 6 months, 9 months and 5 ½ months and 2 ferrets from B&K Marshalls, Male, ages 8 months). Prior to testing sera were heat inactivated at 56°C for 1 hour, receptor destroying enzyme (RDE) treated with 1:10 dilution of the manufacturer’s recommended volume of RDE (Denka Seiken, Japan) overnight at 37°C and heat inactivated for 8 hours at 56°C to remove RDE activity.

### Participating Laboratories for ELLA-NI Testing

7 laboratories participated in the ELLA-NI studies; in alphabetical order, unrelated to the assigned laboratory codes shown in the Figures and Tables: Abbott, NIBSC, Paul Ehrlich Institute, Public Health England, Sanofi Pasteur, University of Bergen, University of Siena. GSK contributed to design of experiments.

### FLUCOP Harmonized ELLA-NI Protocol

Each laboratory received a comprehensive workbook on ELLA-NI testing conditions specific for each study. This described the experimental design for testing linearity, precision, robustness and specificity as well as a detailed SOP for the ELLA-NI including a data reporting template. Sample sera were heat inactivated prior to testing. First, a standard curve of NA activity was carried out for each antigen and used to calculate the dilution required to give 90% of the maximum signal: antigen was serially diluted in PBS and added to a fetuin coated plate. Plates were incubated at 37°C overnight. Plates were washed and lectin from Arachis hypogaea (peanut) (PNA) peroxidase conjugate added and incubated for 120 minutes at room temperature. Plates were washed, TMB added and allowed to develop for 20 minutes then stopped with 0.5M HCl. Plates were read at 450/650mn. The dilution required to give 90% of the maximum signal was calculated. A back titration was carried out to confirm the 90% signal calculation following the procedure described for antigen titration. Once the antigen dilution was confirmed, sera were tested. Serial dilutions of sera and a fixed amount of antigen were mixed before being added to a fetuin coated plate. Plates were incubated at 37°C overnight. Plates were washed, developed and read as described for antigen titration above. The full FLUCOP ELLA-NI SOP can be found in the **Supplementary Material**.

### Statistical Analysis

#### Precision

The coefficients of variation (CV) for repeatability and for intermediate precision were calculated for each sample using a model one-way-ANOVA with the experimental run as a random factor. CV Repeatability (Rep CV) represents the residual variability corresponding to within-run variability. CV Intermediate Precision (IP CV) represents the total assay variability including repeatability and between-run variability. Precision CVs were calculated by sample, by operator for each laboratory on log10-transformed titres. For each lab, a two-way-ANOVA with the sample and the run as random factors was performed on the log10-transformed titres to compute the overall precision using the Mixed procedure of SAS. The acceptance target for functional assays is an IP CV <50%, in line with classical acceptance criteria for neutralisation assays in vaccine licensure.

#### Linearity

Linearity was determined through a dose proportionality approach. The dose proportionality was tested assuming a power model (31), where the logarithm of the measured concentrations is linearly related to the logarithm of the dilutions. This method tests whether the slope of results vs. dilution may be considered equivalent to 1 (dose proportionality was accepted if the ratio (GM_H_/L)/(GM_L_/H) lies within the indicative interval [0.5;2] where H=highest dilution, L=lowest dilution GM=geometric mean). When this is true, linearity is accepted for the whole assay range. When this is false, the range is reduced (lowest value removed, followed if required by the highest value, the two lowest values, the two highest values etc.) and retested until the criterion is satisfied, defining the range for which linearity is accepted.

#### Robustness

Robustness was assessed through an evaluation of end of run effect and an evaluation of three selected parameters on assay performance using a design of experiment (DoE) approach.

#### End of Run

Any samples with values reported as <10 were excluded from analysis. Any samples where values were missing or reported as out of range on plates 1-5 were excluded from analysis. A reference titre for each sample was calculated as the median value from the first 5 plates of the 20 plates in the run. The ratio ‘Result/Median’ was then calculated for each sample and plate. For each plate the overall geometric mean ratio (GMR) across all 6 samples was additionally calculated. GMR is considered acceptable within the indicative interval of [0.8-1.25].

#### Design of Experiment

Two experimental designs were used to assess the effect of incubation time and temperature on assay robustness, based on the ability of laboratories to test two temperatures simultaneously (design 1) or not (design 2). Design 1: For analysis, the reference condition is 37°C, 20 hours virus incubation and 120 minutes PNA incubation and was tested each day. For design 1 firstly day effect was assessed using the GMRs of the reference condition – where a day effect was present, a reference value for GMR calculations was computed by day, where no day effect was observed, replicates across days were used to calculate a reference value. The reference value for a sample was calculated as the geometric mean titre (GMT) of that sample tested in the reference condition. GMR was calculated as the ratio between a sample titre in a given condition and the reference value for that sample.

For design 2 the reference condition was not tested in each run as only 1 temperature can be used for each run. All incubation times were tested each day with different temperatures per day. Here temperature and day are therefore confounded and the effect of temperature cannot be assessed independently of day effect. The impact of temperature and incubation times is described using two reference values:

i) reference value is the GMT of the sample tested at 37°C, 20h virus incubation and 120 minutes PNA incubation. Here the effect of temperature is assessed (however it should be noted that a random day effect cannot be excluded)
ii) reference value is the GMT of the sample tested at 20 hours virus incubation and 120 minutes PNA incubation by temperature and day (i.e. a different reference value for 36°C, 37°C and 38°C for each day). Here the effect of incubation times is assessed.

GMR was calculated as the ratio between a sample titre in a given condition and the appropriate reference value for that sample. GMR is considered acceptable within the indicative interval of [0.8-1.25].

#### Calibration of ELLA-NI Titres

Three mid-range samples were selected as calibrators; sample 6 for the precision data, sample 10 for robustness data and sample 20 V2 (donor 20, visit 2 (V2) post vaccination) for the testing of B virus strains and rNA antigen. For each data set a calibration factor was calculated as the ratio of the calibrator titre in an ELLA-NI run/the global GMT of the calibrator sample (GMT of all times the calibrator sample was tested across all participating laboratories). The calibration factor was then applied to other titres within that lab, run and repeat. The GMR was calculated as the GMT of the lab/overall GMT of a sample across all labs. GMR was calculated before and after calibration. For the precision dataset, using a mixed approach of SAS, a 2-way ANOVA with the sample and lab as random factors was performed on log_10_ transformed calibrated titres to calculate intra-lab %CV and Reproducibility %CV (intra-lab and inter-lab variation combined). For each data set, %GCV (Geometric Coefficient of Variation) across all labs for a sample was calculated as (10^s^-1)x100%, where s is the standard deviation of the log_10_ titres. %GCV was calculated before and after calibration (‘overall %GCV’ is the median %GCV across all samples in a panel), and the change %GCV statistically assessed using the Wilcoxon matched pairs test.

## Results

### HA Antibody Interference With ELLA-NI and the Use of Appropriate Antigen

An HA competition analysis was carried out to confirm the role of anti-HA antibodies in false positive ELLA-NI titres. Three mouse sera were tested: H1N1 vaccinated (positive for H1 and N1 antibodies), recombinant HA (rHA) inoculated (positive for H1 antibodies) and PBS inoculated (negative for H1 and N1 antibodies). Sera were pre-incubated with either a rHA from A/California/07/2009 virus (H1N1) or two irrelevant proteins (glycoprotein B (gB) protein from cytomegalovirus (CMV) and non-structural-1 (NS1) protein from Japanese encephalitis virus (JEV)) before titration in ELLA-NI using A/California/07/2009 virus (H1N1).

**Figure 1:**
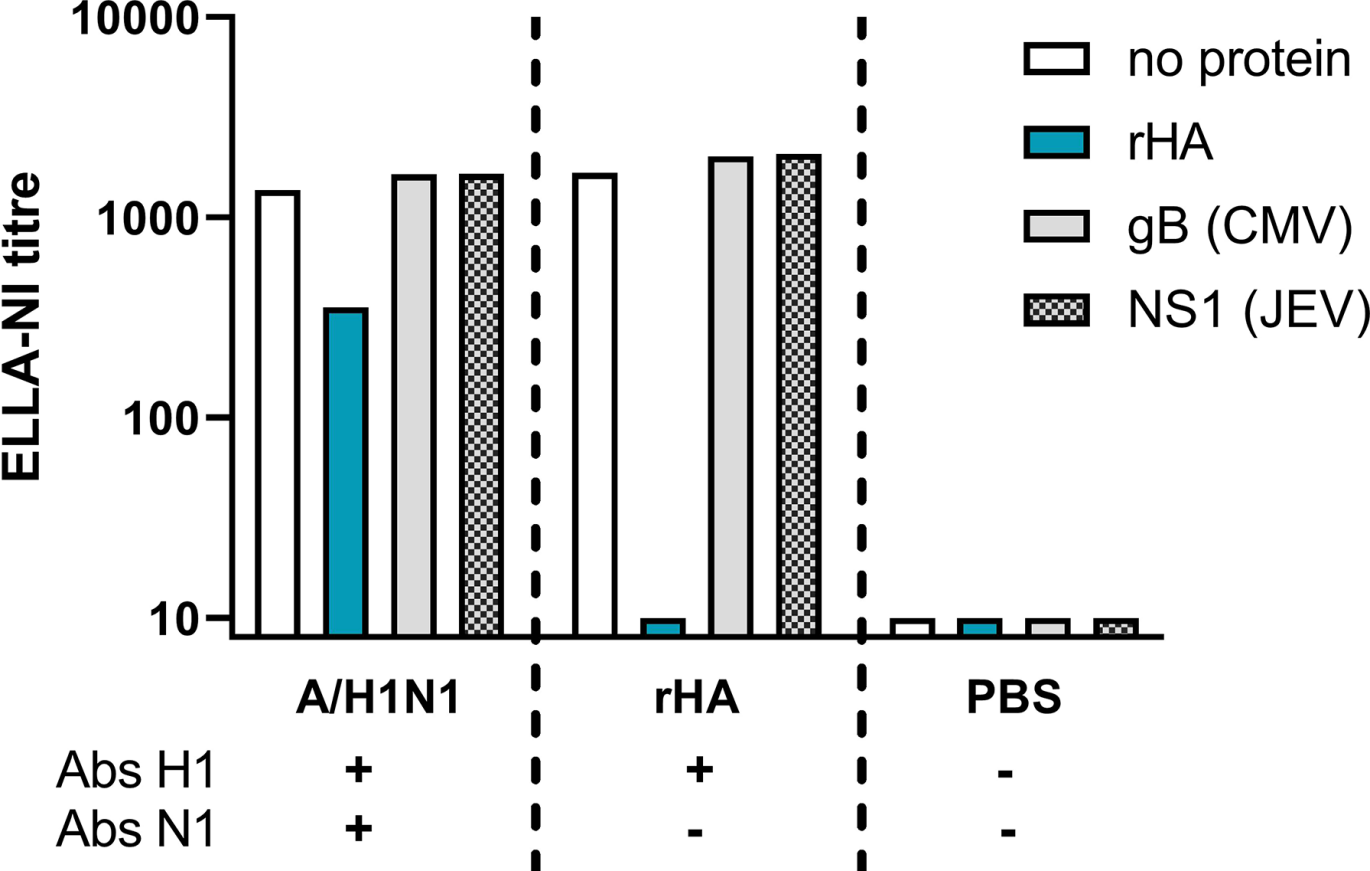
Competitive binding of anti-HA antibodies prior to ELLA-NI testing. Sera from mice vaccinated with A/H1N1pdm09 (A/California/07/2009) monovalent vaccine, rHA (from the same strain) or PBS were pre-incubated either with no protein, with rHA (A/California/07/2009) or one of two irrelevant viral proteins (gB from CMV or NS1 from JEV). Pre-incubated sera were then tested in ELLA-NI with a live H1N1 A/California/07/2009 virus. Sera from H1N1 vaccinated mice contained specific antibodies against both H1 and N1, while sera from rHA vaccinated mice were antibody positive for H1 only and sera from PBS vaccinated mice were antibody negative for both H1 and N1.

In the presence of both anti-HA and anti-NA antibodies, a 74% reduction in ELLA-NI titre was observed when anti-HA antibodies were competitively bound to rHA (see **Figure 1**). This reduction was specific to incubation with rHA and absent when irrelevant proteins were used. When only anti-HA antibodies were present, competitive binding with rHA abolished ELLA-NI titre. These results demonstrate the role of HA antibodies in the overestimation of NI titres in this assay, confirming the need to either: use reverse genetics virus with an HA not in circulation in humans; remove HA specific antibodies in sera prior to testing; or use an alternative source of NA (for example rNA or lentiviral pseudotypes).

### Validation of a Consensus ELLA-NI Standard Operating Procedure (SOP)

We carried out a review of ELLA-NI protocols used by laboratories within the FLUCOP consortium and developed a consensus SOP based on commonality between protocols, taking into account lab-specific limitations and recommendations based on previous publications optimising ELLA-NI. This detailed SOP can be found in the **Supplementary Material**.

Seven laboratories from the FLUCOP consortium participated in a validation of the ELLA-NI SOP, testing precision, linearity, robustness, and specificity in line with classical acceptance criteria for neutralisation assays used for vaccine evaluation (see materials and methods for experimental design, statistical analysis and acceptance criteria. All testing laboratories used an RG virus containing the N1 NA of the A/California/09/2009 virus with either H7 or H9 (see **Table 1**) for precision, linearity and robustness).

### Precision of ELLA-NI

A precision analysis was carried out testing a panel of 9 positive samples spanning the analytical range of the assay. Each sample was tested in duplicate on the same plate, in parallel on a separate plate (giving 2 repeats/operator/run) and by a second operator (Series 1 and 2) on four different days generating up to 16 titres per sample for series 1 and series 2. Six labs participated (5 labs returned data for 2 operators and 1 lab returned data for 1 operator).

Repeatability (Rep) %CV (residual variability corresponding to *within*-run variability) and Intermediate Precision (IP) %CV (total assay variability including repeatability and *between*-run variability designed to mimic routine assay runs) were calculated (see materials and methods and **Table S1**).

For each laboratory the overall IP precision was calculated and was considered acceptable (aiming for an IP %CV <50% for functional assays) ranging from 7.6-34.6% (see **Table 2**). Testing samples in duplicate did decrease IP %CV, however the improvement was small (see **Table S2**) and no clear difference in intra- or inter-plate duplicate IP CVs was observed. Consequently, the routine testing in singleton, where sera volumes are small or to increase throughput, was considered acceptable. Precision by operator was comparable for most laboratories, with some small differences between laboratories in Rep and IP %CV, however IP %CV was still less than 37% across operators and laboratories, below the acceptance target for functional assays of 50% (see **Supplementary Table S3**).

**Table 2:** Overall precision analysis - intermediate precision (IP) and repeatability (Rep) %CV per laboratory (acceptance target of IP CV < 50% for functional assays).

			Series 1		Series 2		Precision range	
Lab	N samples	N results used	RepCV (%)	IPCV (%)	RepCV (%)	IPCV (%)	LLP	ULP
1	9	144	10.4	31.6	15.8	34.4	27.1	4855.5
2	8	128*	14.9	24.7	15.4	27.8	30.4	2010.9
3	9	144	10.7	20.3	10.6	15.5	63.8	7555.7
4	9	144	20.2	34.4	20.0	34.6	32.8	7023.5
5	9	72**	9.7	19.6	8.9	16.1	52.3	6901.9
7	9	144	5.5	7.7	4.7	7.6	11.4	3733.9

* Lab 2 returned titres of < 10 for a sample and this sample was excluded from analysis.

**Lab 5 returned data from 1 operator only.

The precision range, delimited by the Lower Limit of Precision (LLP) and the Upper Limit of Precision (ULP), was determined as the range of titres where the IP CV (%) estimated is lower than 50% for each laboratory (see **Table 2**).

### ELLA-NI Is Linear Across a Large Titre Range in All Testing Laboratories

An assessment of dilutional linearity was carried out using 4 serum samples diluted ½, ¼ and ⅛ in a negative matrix (an Ig depleted serum – Sigma S5393). Each fractional dilution was carried out independently. Undiluted sera and the three fractional dilutions were tested in 8 runs (4 repeats by 2 operators), except for Lab 5 (4 repeats by 1 operator). All dilutions for a single serum sample were run on the same plate. Linearity was determined through a dose proportionality approach (see materials and methods). All 6 labs demonstrated linearity across the range of samples tested giving the lower limit of linearity (LLL) and the upper limit of linearity (ULL) for each laboratory (see **Table 3**).

**Table 3:** Summary of LLOQ and ULOQ for each testing laboratory.

Lab	LLP	ULP	LLL	ULL	LLOQ	ULOQ
1	27.1	4855.5	31.4	4202.8	32	4202
2	30.4	2010.9	23.9	1846.9	31	1846
3	63.8	7555.7	62.2	6252.8	64	6252
4	32.8	7023.5	41.9	2238.7	42	2238
5	52.3	6901.9	18.0	6504.1	53	6504
7	11.4	3733.9	41.5	3727.3	42	3727

Lower and upper limits of precision (LLP and ULP), lower and upper limits of linearity (LLL and ULL), and lower and upper limits of quantitation (LLOQ and ULOQ) are shown for each laboratory. LLOQ and ULOQ define the range in which the FLUCOP ELLA-NI SOP delivers both precision and linear accuracy for the testing laboratories.

### Limits of Quantitation

Using the linearity and precision profiles of each laboratory, the limits of quantitation can be defined: the lower limit of quantitation (LLOQ) is the higher value between LLL and LLP, and the upper limit of quantitation (ULOQ) is the lower value between ULL and ULP. **Table 3** gives the LLOQ and ULOQ of the six testing laboratories. LLOQ is consistent between laboratories (min-max 31-64), ULOQ is more variable from lab to lab (min-max 1846-6504) however a large range of titres are within these limits for all testing laboratories.

### ELLA-NI Robustness: End of Run Analysis

**Figure 2:**
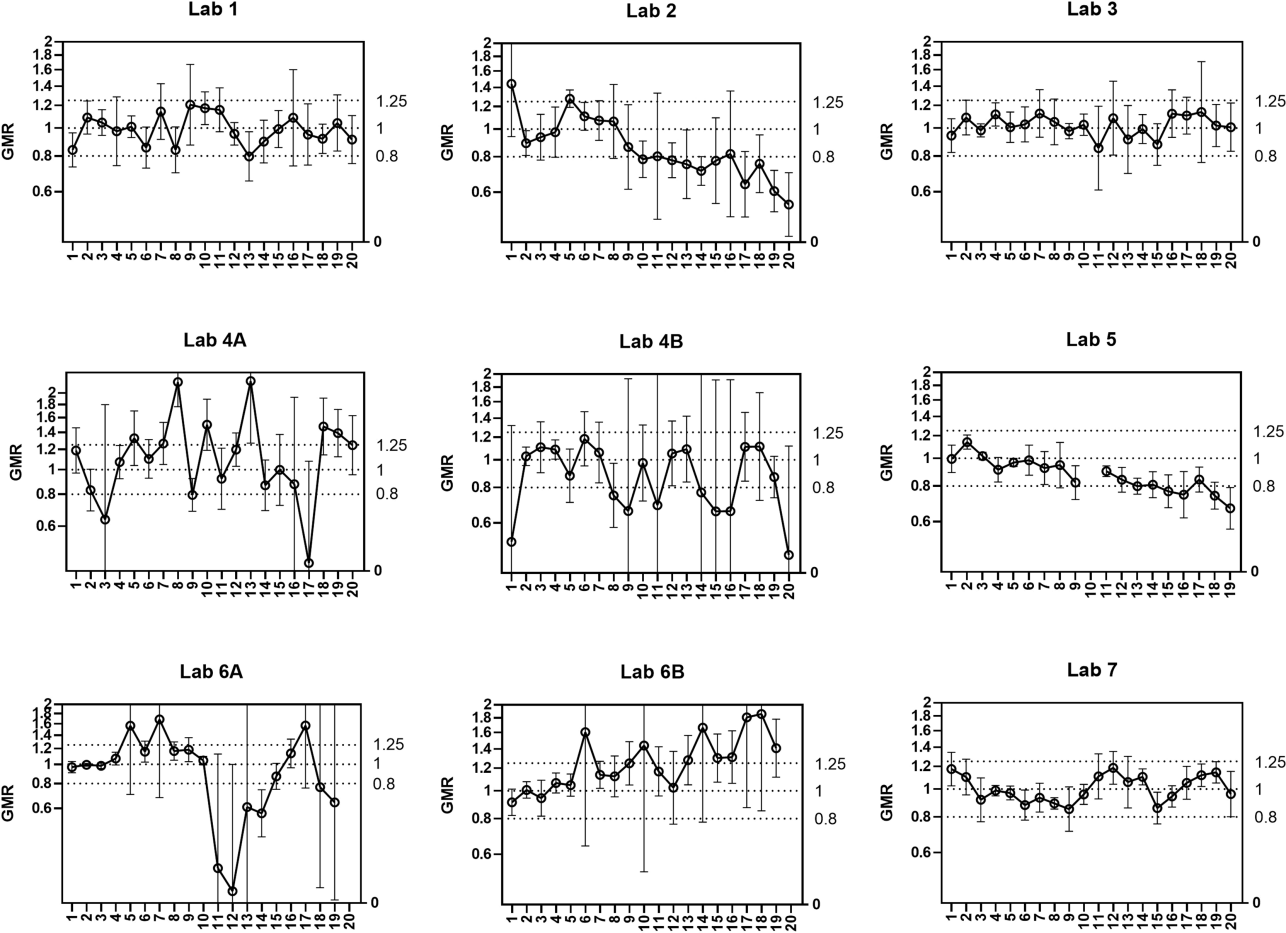
Within-run assay performance – number of plates per run (end of run). 6 samples were tested per plate for 20 plates in a single run. Seven participating laboratories returned data (two labs returned data for 2 operators - A/B) carrying out a single run. A reference titre for each sample was calculated as the median from plates 1-5 for geometric mean ratio (GMR) calculation. The geometric mean of GMR per plate is plotted over 20 plates (error bars show 95%CI). Indicative interval of 0.8-1.25 is shown by dashed lines. After data exclusion as in material and methods Lab1 N=6, Lab 2 N=6, Lab 3 N=5, Lab 4 N=6, Lab 5 N=4, Lab 6 N=6, Lab 7 = 5.

End of run analysis was designed to identify the maximum number of plates that can be tested in a single assay run. The same set of 6 samples was tested on 20 plates in a single run. Seven laboratories took part in the testing. By laboratory, a reference titre for each sample was calculated as the median titre of the first 5 plates. The ratio ‘Result/Median’ was calculated for each sample on each plate, and then the geometric mean ratio (GMR) of all samples on one plate was calculated. We expect the GMR of each plate to fall within the indicative range [0.80-1.25]. **Figure 2** plots the GMRs for 7 participating laboratories; two labs provided data for two operators (A and B). Laboratories 1, 3 and 7 had consistent GMRs, however laboratories 2 and 5 showed a systematic bias with GMR decreasing over the 20 plates, laboratory 6A showed a systematic increase in GMR and laboratories 6 and 4 showed an increase in variability in GMR as the number of plates increases. As a conclusion from these results we recommend a limit of 10 plates per run to avoid systematic bias and reduce within-run variability. It should be noted that we did not investigate the impact of including a calibrator on each plate within a run: it is possible that a greater number of plates could be run using this approach. The recommendation of a 10-plate limit applies where a calibrator is not included on each plate.

### ELLA-NI Robustness: Impact of Incubation Times and Temperature

**Figure 3:**
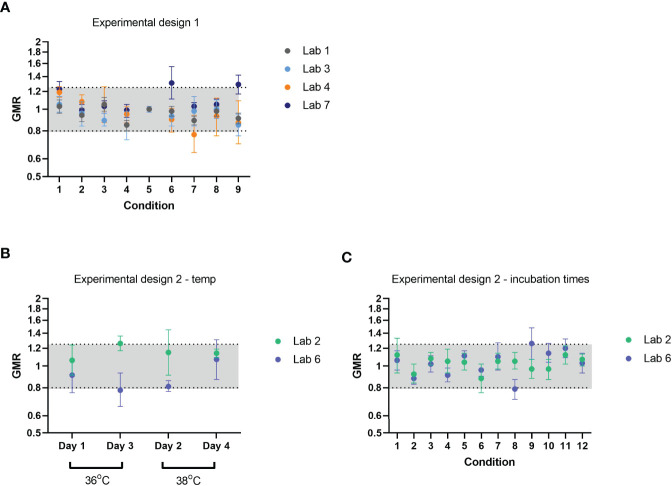
Robustness of FLUCOP ELLA-NI SOP; varying incubation time and temperature. Geometric mean ratios (GMR) per condition across 12 samples are plotted for each testing laboratory. Conditions tested are varying combinations of virus incubation temperature (37°C +/- 1 degree ─ or +/- 2 degrees for Lab 1), virus incubation time (20h +/- 1h) and PNA incubation time (105/120/135 minutes) according to a design of experiment (see **Table 4**). Two experimental designs were carried out based on the laboratories’ ability to test multiple temperatures in a single ELLA run **(A)** or a single temperature per run **(B, C)**. Different statistical analysis approaches were used for the different designs to allow for day effect and confounding factors. GMRs were calculated relative to an appropriate reference value for each sample, for each design, as described in materials and methods. Upper and lower 95% CI are shown as error bars. The indicative range of 0.8-1.25 is shaded in light grey.

Variation in three main parameters was assessed for impact on ELLA-NI titres: virus incubation time (20h +/-1h), virus incubation temperature (37°C +/- 1 degree ─ or +/- 2 degrees for Lab 1) and PNA incubation time (120 min +/- 15 mins). Laboratories tested 12 samples using two different experimental designs depending on the testing capability of each laboratory. Experimental design 1 was carried out where multiple temperatures could be tested within a single ELLA run (see **Table 4** for experimental design). GMR was calculated using the appropriate reference value for each laboratory for each condition (see materials and methods). Almost all GMRs fell within the indicative interval [0.8-1.25] and no specific condition was associated with changes in GMR (see **Figure 3A**). Experimental design 2 was carried out where a single temperature could be used per ELLA run (see **Table 4** for experimental design). To assess the impact of temperature (**Figure 3B**) and virus and PNA incubation times (**Figure 3C**), appropriate reference values for each sample were computed (see materials and methods) and GMR per lab per condition calculated. As for experimental design 1, almost all GMRs fell within the indicative range [0.8-1.25] and no specific condition was associated with changes in GMR. These data indicate that the assay was robust within the following tolerances: virus incubation temperature 37+/-1/2°C, virus incubation time 20 hours +/-1h and PNA incubation time 120 minutes +/-15 minutes.

**Table 4:** Experimental conditions for design 1 and design 2 to assess robustness of ELLA-NI.

Experimental design 1			
Condition	Temp (°C)	O/N incubation (hours)	PNA incubation (minutes)
1	(35*)-36	19	105
2	(35*)-36	19	135
3	(35*)-36	21	105
4	(35*)-36	21	135
5	37	20	120
6	38-(39*)	19	105
7	38-(39*)	19	135
8	38-(39*)	21	105
9	38-(39*)	21	135
**Experimental design 2**			
**Condition**	**Temp (°C)**	**O/N incubation (hours)**	**PNA incubation (minutes)**
1	36	19	105
2	36	19	135
3	36	21	105
4	36	21	135
5	37	19	105
6	37	19	135
7	37	21	105
8	37	21	135
9	38	19	105
10	38	19	135
11	38	21	105
12	38	21	135

*Lab 1 tested a variation of +/- 2 degrees.

### Intra-Laboratory Variability in ELLA-NI Is Comparable Across Influenza A and B Viral Antigens

**Figure 4:**
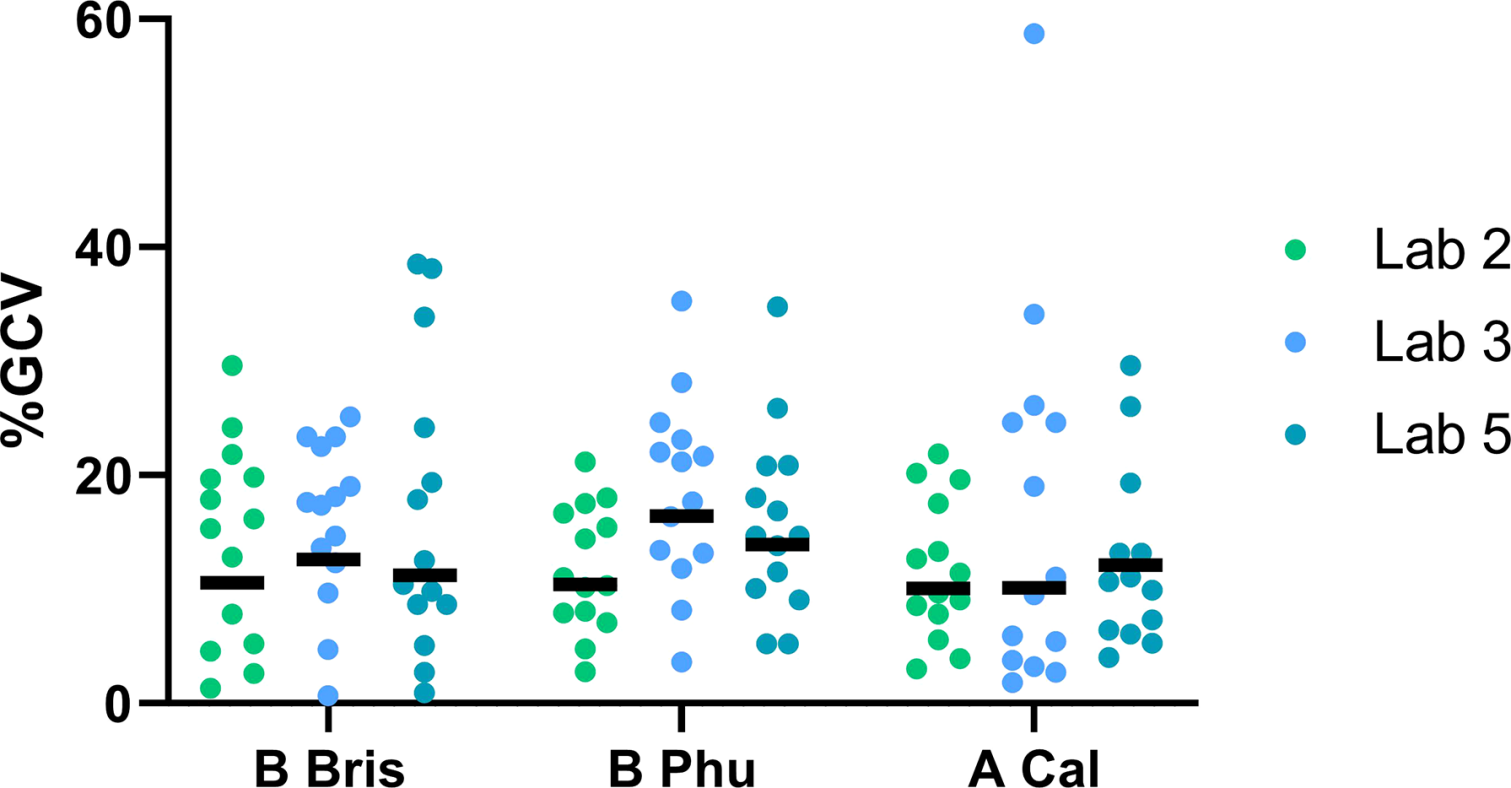
Intra-laboratory performance using three influenza strains is consistent. Three mismatched influenza viruses were tested using the FLUCOP ELLA-NI SOP. A serum panel of 16 human sera was tested in three laboratories. Each laboratory carried out three independent runs. %GCV per sample is shown by lab for: B Bris (H9 with B/Brisbane/60/2008 NA), B Phu (H9 with B/Phuket/3073/2013 NA) and A Cal (H7N1 or H9N1 with A/California/07/2009 NA). The geometric mean of %GCV is shown as a black bar (negative samples 12 and Ig- were excluded from analysis).

Two RG influenza viruses were generated containing the NA of B/Brisbane/60/2008 and B/Phuket/3073/2013 (both contain an influenza A H9 HA, see materials and methods and **Supplementary Figure S1**). This allowed us to test mismatched influenza A and B (both Yamagata and Victoria lineage) viruses side by side for the first time. Three laboratories tested three RG viruses: H9 with B/Brisbane/60/2009 NA; H9 with B/Phuket/3073/2013 NA; H7 or H9 with A/California/07/2009 NA against a panel of 16 human sera samples. Laboratory performance for all three virus strains tested was comparable to results from previous testing with N1 alone (see **Figure 4**). Overall %GCVs for samples tested with both B strain viruses were acceptable ranging from 15.8-17.4 for B/Brisbane/60/08 and 15.9-18.6 for B/Phuket/3073/13. Variation in %GCV was uniform across the sample panel tested (see **Supplementary Figure S2**) with no strain specific differences. These results demonstrate the consistent performance of the Flucop ELLA-NI SOP with multiple RG mismatched viral antigens including influenza B viruses.

### ELLA-NI Demonstrates Specificity for Different Influenza Types and B Lineages

Specificity studies for influenza serology assays cannot be carried out using human sera, as individuals have a complex immunological history of exposure to multiple influenza strains or vaccines. To overcome this, monospecific ferret sera were used to test the specificity of the ELLA-NI. These ferrets were exposed to single strain of influenza and are negative for antibodies against other influenza strains. Sera tested were from ferrets infected with a) B/Brisbane/60/2008 WT virus (B/Victoria lineage) (2 individual ferrets), b) B/Phuket/3073/2013 WT virus (B/Yamagata lineage) (2 individual ferrets) and c) A/California/07/2009 (H1N1)pdm09 WT virus (2 individual ferrets). These viruses have the same NA as the antigens used in the ELLA-NI. Preliminary studies showed that sera require RDE treatment to remove non-specific inhibitors of NA activity. In agreement with previously published data, RDE diluted to 1:10 was sufficient to remove non-specific inhibitors and a longer 8 hours heat inactivation at 56°C was required to remove all RDE activity (20). Ferret sera were pre-screened using HAI to confirm the absence of anti-H9 or anti-H7 antibodies that could cause non-specific NA inhibition (data not shown). Ferret sera were tested in both homologous (NA serum raised against the same virus as the test antigen) and heterologous (NA serum raised against a virus different from the test antigen) pairs. Type specificity was clear, and ELLA-NI also differentiated between the B Yamagata and B Victoria lineage viruses (see **Supplementary Figure S3**). NI titres for homologous NA/anti-sera pairs were high and reproducible for all three influenza virus strains tested (titres for the 2 individual ferrets for B/Phuket were 2643/4137; B/Brisbane were 2942/3196 and A/California were 1561/1823). Heterologous NA/anti-sera NI titres were very low or negative (range of 5-31, GMT of 8.5).

### The Use of a Mid-Titre Human Serum as a Calibrator Improves Inter-Laboratory Agreement

**Figure 5:**
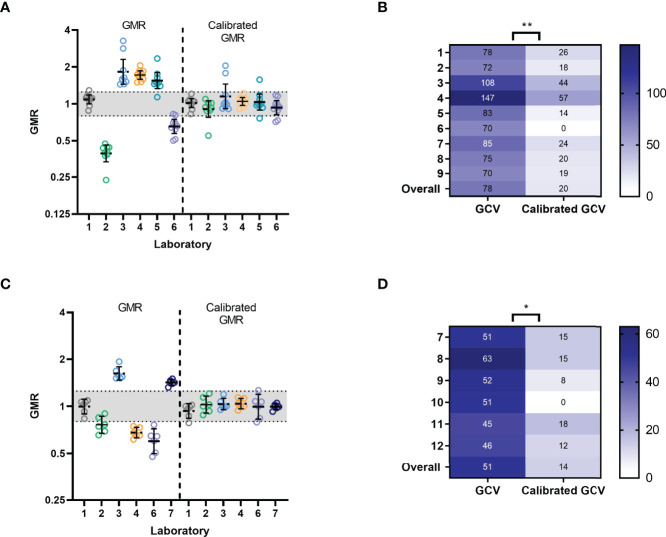
Calibration of Precision data and Robustness data**: (A)** Precision data: geometric mean ratios (GMR) for uncalibrated and calibrated ELLA-NI titres by lab per run. For each laboratory and run, a calibration factor was calculated using sample 6 (see materials and methods, sample with a mid-range titre) and applied to titres. The indicative range of 0.8-1.25 is shaded in grey. Geometric mean and 95% CI error bars are shown in black. **(B)** Precision data: %GCV for log_10_ transformed titres before and after calibration with sample 6. ** indicates significance using the Wilcoxon matched pairs test [P=0.0039] **(C)** Robustness data: GMR of ELLA-NI titres for samples 7-12 are plotted before and after calibration with serum sample 10 (selected as a sample with a mid-range titre). The indicative range of 0.8-1.25 is shaded in grey. Geometric mean and 95% CI error bars are shown in black. **(D)** Robustness data: %GCV for log_10_ transformed titres before and after calibration with sample 10. * indicates significance using the Wilcoxon matched pairs test [P=0.0313].

Using the data obtained in our precision analysis, we selected serum sample 6 (having mid-range titres in all testing laboratories) to calibrate our precision results. A per run calibration factor was calculated and applied to titres. After calibration, the GMR for each sample was calculated (sample GMT by lab/overall GMT across all labs). As expected, the GMRs of calibrated titres were closer to 1 than those of un-calibrated titres, and %GCV was significantly reduced for all samples tested (see **Figures 5A, B**). We used calibrated titres to calculate the Intra-lab CV (reflecting within-lab variability), inter-lab CV and Reproducibility CV (reflecting the total inter-laboratory variation including intra-lab variation). **Table 5** shows the Intra-lab CV, Inter-Lab CV and Reproducibility CV before and after calibration per lab, run and repeat with mid-titre sample 6. The use of a calibrator had little impact on intra-laboratory variation as expected, but substantially reduced inter-laboratory variability with Reproducibility CV reducing from 73% to 30%. These data clearly demonstrate the benefit of using a calibrator to reduce inter-laboratory variation when testing an N1 virus in ELLA-NI. We repeated this analysis using data obtained in the robustness study. Comparison of GMR and %GCV for titres obtained for samples 7-12, tested using the FLUCOP SOP, before and after calibration with mid-range titre serum 10 showed reductions of GMR and %GCV (**Figures 5C, D**).

**Table 5:** Intra-lab CV, Inter-lab CV and Reproducibility CV for uncalibrated titres and calibrated titres by lab, run and repeat.

	Titres Intra-lab CV (%)	Inter-lab CV (%)	Reproducibility CV (Intra-lab and Inter-lab) (%)
**Uncalibrated**	24.73	66.48	72.81
**Calibrated by lab, run and repeat**	23.87	18.45	30.49

**Figure 6:**
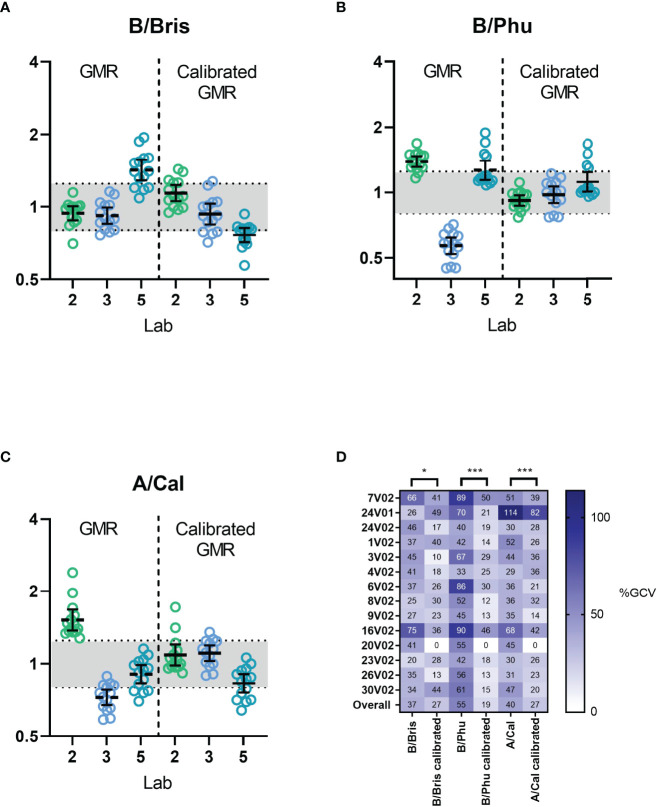
Inter-laboratory performance before and after calibration. Three reverse genetics (RG) influenza viruses, **(A)** H9 with B/Brisbane/60/2008 NA, **(B)** H9 with B/Phuket/3073/2013 NA and **(C)** H7N1 with A/California/07/2009 NA, were tested using the FLUCOP ELLA-NI SOP. Geometric mean ratios (GMR) of ELLA-NI titres are shown before (left) and after (right) calibration using serum sample 20 V02 as a calibrator. The indicative range of [0.8-1.25] is shaded in light grey. The geometric mean and 95% CI error bars are plotted in black. **(D)** %GCV per sample is shown before and after calibration (negative samples 7V01 and Ig- were excluded from analysis). * indicates statistical significance using the Wilcoxon matched pairs test [* P=0.0494, ***P=0.0001 and ***P=0.0009 respectively].

We next assessed the use of a calibrator using the data from testing of multiple influenza strains. We selected serum sample 20 V02 (having mid-range ELLA-NI titres for B/Brisbane/60/2008, B/Phuket/3073/2012 and A/California/07/2009 RG viruses) as a calibrator. **Figure 6** shows the GMR of ELLA-NI titres by virus strain before and after calibration (**Figures 6A–C**) and inter-laboratory %GCV before and after calibration for each serum sample (**Figure 6D**). A significant improvement in inter-laboratory agreement could be seen after calibration as GMRs were closer to 1, with most values falling within the indicative range [0.8-1.25]. Overall %GCV was significantly reduced for all three virus strains (from 37% to 27% for B/Brisbane60/2008, from 55% to 19% for B/Phuket/3073/2013 and from 40% to 27% for A/California/07/2009). Together these data clearly show the benefit of using a calibrator to reduce inter-laboratory variation in multiple independent studies testing three different strains of influenza.

The distribution of ELLA-NI titres before and after calibration, along with two pre-(V01)/post-(V02) vaccinations pairs included in the serum panel can be seen in **Supplementary Figure S4**.

### Evaluation of N1, N2 and B rNA as Source of Antigen in ELLA-NI

**Figure 7:**
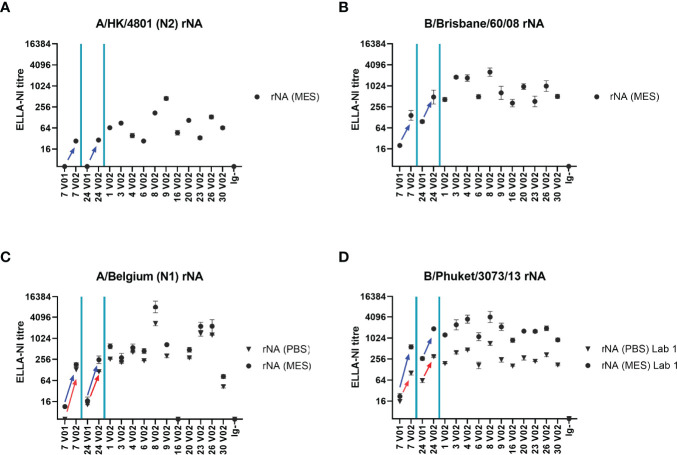
Recombinant NA (rNA) antigen in ELLA-NI. A panel of 16 human sera were titrated in ELLA-NI using rNA from **(A)** A/Hong Kong/4801/2014 (N2) **(B)** B/Brisbane/60/2008 **(C)** A/Belgium/145-MA/2009 (N1) and **(D)** B/Phuket/3073/2013 using either MES (circles, all four rNAs tested) or PBS (triangles, A/Belgium and B/Phuket only) based-buffers. Paired pre- (V1) and post- (V2) vaccination sera from two individuals (7 and 24) were included in the serum panel. Increases in ELLA-NI titres after vaccination are indicated by arrows (blue for MES buffer and red for PBS buffer). GMT of 2-4 independent titrations is plotted with geometric SD error bars.

We finally evaluated the use of rNA in ELLA-NI using the same 16 sample serum panel tested with mismatched RG N1 and B viruses. Sera were titrated in ELLA-NI against rNAs from 4 influenza strains: A/Hong Kong/4801/2014 (N2), B/Brisbane/60/2008, A/Belgium/145-MA/2009 (N1, A/California/07/2009-like) and B/Phuket/3073/2013, using an MES based buffer. **Figure 7** shows ELLA-NI GMTs. A/Belgium/145-MA/2009 and B/Phuket/3073/2013 were additionally tested using a PBS-based buffer (see **Figures 7C, D**). Titres measured using an MES-based buffer were consistently higher than those measured using a PBS-based buffer: the difference was more pronounced with the rNA from B/Phuket/3073/2013 (**Figure 7D**), with increases ranging from 1.5- to 8.6-fold, than with the rNA from A/Belgium/145-MA/2009 (**Figure 7C**), with increases ranging from 1.3 to 3.0-fold. This strain-strain variation was likely due to differences in NA activity at low (MES buffer) versus neutral (PBS buffer) pH consistent with previous observations (18). As expected, for the two volunteers for which paired sera (pre-and post-vaccination) were available (7 V1/2 and 24 V1/2), titres were increased after vaccination with all the tested antigens. The titre of the Ig depleted human serum negative control was found to be below the detection level in all the tested conditions.

### Calibration Using a Mid-Titre Serum Sample Improves Agreement in ELLA-NI Titres Between rNA and RG Virus Antigens

As we previously tested the NA from B/Phuket/3073/2013 both as a mismatched RG virus and as an rNA using the same serum panel, we were able to assess the impact of calibration on agreement in NI titres using these different sources of antigen.

**Figure 8:**
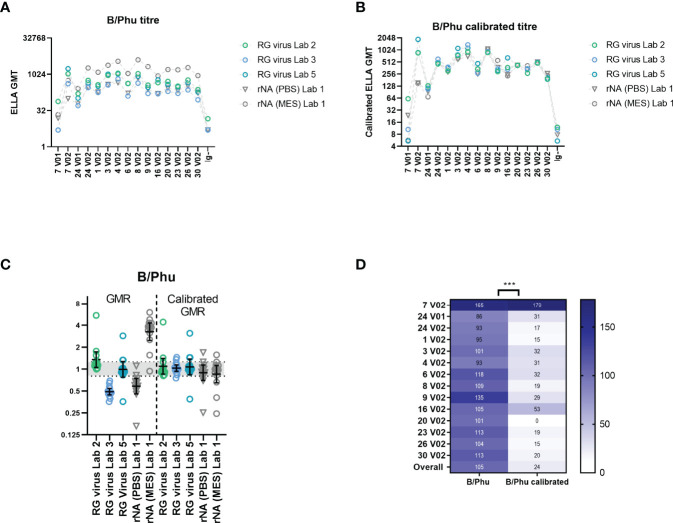
Comparison of B/Phuket/3073/2013 rNA and mismatched HA/NA reverse genetics (RG) viruses in ELLA-NI and calibration using a mid-titre serum sample. **(A)** ELLA-NI titres for B/Phuket/3073/2013 (B/Phu) RG virus tested in three labs (Labs 2/3/5) and rNA from B/Phuket/3073/2013 tested in one lab with either a DPBS buffer [rNA (PBS)] or an MES buffer [rNA (MES)]. **(B)** ELLA titres as in [A] after calibration with mid-titre serum sample 20 V2. **(C)** Geometric mean ratios (GMR) of B/Phuket/3073/2013 RG virus and rNA ELLA-NI titres before and after calibration. The geometric mean and 95% CI are shown in black **(D)** %GCV per sample across the 5 labs/methods before and after calibration. *** indicates significance using the Wilcoxon matched pairs test [P=0.0002].

**Figure 8A** shows the GMTs of B/Phuket/3073/2013 tested in three laboratories using mismatched RG virus (Lab 2/3/5) and the GMTs of rNA B/Phuket/3073/2013 tested in laboratory 1 using PBS buffer and MES buffer. All testing laboratories and methods give the same trend for all samples tested, however rNA NI titres when using MES buffer were substantially higher than those obtained using PBS buffer or using RG viruses.

Following the same approach applied to our previous data sets, calibration using the mid-titre sample 20 V2 improved inter-laboratory agreement. **Figure 8B** shows the GMTs for each sample tested with B/Phuket antigens after calibration, with closer agreement between different testing laboratories and antigens. **Figure 8C** shows the GMRs across all samples tested before and after calibration, with GMR becoming much closer to 1 (particularly evident for rNA testing using MES buffer), with the majority of GMR values falling within the indicative range of [0.8-1.25] (37% of GMR values fall within this range before calibration, rising to 77% after calibration). **Figure 8D** shows the %GCV before and after calibration, again showing a significant improvement in agreement between laboratories.

The same approach was also applied to ELLA-NI titres obtained using A/Belgium/145-MA/2009 (A/California/07/2009-like) rNA and ELLA-NI titres obtained using A/California/07/2009 NA mismatched RG virus (see **Supplementary Figure S5**). Calibration using mid-titre sample 20-V2 improved inter-laboratory agreement, with GMRs closer to 1 and %GCV decreased for the majority of samples.

These data show that tetrabrachion rNA could be used as an antigen in ELLA-NI testing as an alternative to RG influenza viruses; however when testing with MES buffer, ELLA titres could be substantially higher than observed when testing with RG viruses and show strain to strain variability. We show that a calibrator could be used to address this problem, highlighting the importance of developing standards for seasonal influenza serology.

## Discussion

FLUCOP aims to provide a toolbox of serological assays for influenza through two mains methods i) encouraging the use of consensus, harmonized SOPs that have been assessed in international collaborative studies, and ii) investigating the applicability and relevance of potential serology standards. To improve harmonization and inter-laboratory agreement of serological testing (essential for assays used to define correlates of protection), these assays need to be precise, robust, and in the case of influenza must also have minimal strain-strain variability in performance whilst additionally being able to differentiate between subtypes. As interest in NA as a target antigen for novel vaccines grows, standardized methods for testing functional anti-NA antibodies would greatly facilitate development and regulation of these novel vaccines. ELLA-NI has already been used for studying anti-NA antibody responses post vaccination (32) and in clinical trials (29). Building upon previous studies optimising ELLA-NI (19, 20) and studies showing ELLA-NI to be robust within a single laboratory setting (19, 20, 33), here we provide a detailed harmonized SOP (see **Supplementary Material**) demonstrated through our international collaborative studies to be precise, linear and robust when testing using N1 antigen.

Two essential attributes for influenza serology assays are strain-strain consistency in performance and ability to differentiate between influenza subtypes. Previous studies using ELLA-NI to detect antibodies to B influenza viruses have used WT viral antigens, where contribution of anti-HA antibodies to B virus NI titres could not be excluded (19). In this study we tested the specificity of two mismatched RG viruses with the NA from B Victoria and B Yamagata lineage viruses and the HA from an A/H9 virus for the first time. A previous study developed an H6 RG B Yamagata NA containing virus using a very similar approach to that used here (28) and demonstrated the virus performed well in ELLA studies. These data support the use of ELLA-NI for detection of both A and B influenza NA antibodies with consistent assay performance, and importantly differentiation between B Victoria and B Yamagata lineages with almost no cross reactivity. It would be interesting to test these mismatched RG B viruses with sera from previous or subsequent vaccination campaign years to assess how antigenic drift is captured by ELLA-NI. A previous study tested H1N1/H3N2 NA drift using the TBA assay (34) and H3N2/H2N2 NA antigens in ELLA-NI spanning 5 decades, demonstrating that drifted variants can be detected with ELLA-NI (20), but this remains to be shown for both N1 (in ELLA-NI) and B antigens.

Inter-laboratory agreement can be improved both by using harmonized protocols and by using a biological standard, allowing for the normalization and direct comparison of assay results regardless of the protocol used to derive them. Seasonal influenza presents a particular problem in regard to biological standards, as levels of antigenic drift are high (35). Different strains within a subtype will change antigenically over time, making definition of international units against individual strains difficult and impacting on the useable lifespan of a seasonal influenza biological standard. Nevertheless, standards have been proven to reduce inter-laboratory variation using HAI, Virus Neutralisation (VN) and ELLA-NI (21, 36–40) and warrant further development. In the absence of commercially available biological standards, we selected a mid-titre human serum sample from each panel tested as a calibrator. One previous study has looked at the inter-laboratory performance of ELLA using less strict harmonization criteria for testing than used in this study (21). An overall %GCV of 112% was calculated for N1 antigen (falling to 59% after normalization with a standard). Our use of a detailed harmonized SOP along with harmonized data analysis appears to give lower variation than previously observed (overall %GCV for our precision data of 78% falling to 20% after calibration with a standard), however it should be noted that a greater number of labs participated in that previous study, leading perhaps to increased variability. Acceptance criteria for background levels and titration of antigen were identified as critical for improved inter-laboratory agreement (21) – both these considerations have been included in the harmonized SOP presented here. Our use of a calibrator clearly and significantly reduced inter-laboratory variation in every collaborative study carried out, demonstrating the consistent improvement across studies using multiple influenza strains. The continued development of seasonal standards for influenza remains a priority. Work is also required to investigate the best source of a biological standard. Pools of human sera were used in this study, where large volumes can be created through pooling of multiple donors. Other potential sources include antisera from animals – this approach has the added advantage of inoculating animals with recombinant NA to avoid any interference of anti-HA antibodies. However the ethical burden of this approach must be taken into consideration. It will be important to investigate the possible lifespan of a standard. The fact that NA experiences slower antigenic drift than HA (9) may be advantageous and prolong the lifespan of a seasonal standard for NA.

One drawback to using ELLA is the interference of anti-HA antibodies in the assay. In this study we initially confirmed the specific role of HA antibodies in generating false positive titres, or in overestimating NI titres. This is in agreement with other studies using both ELLA and the TBAA (19, 24, 41). Kosik et al. show that HA-specific mAbs can inhibit NA activity only when HA is in close proximity to NA (an effect abrogated by detergent disruption of virions) (24), and they suggest two mechanisms through which HA antibodies interfere with ELLA: firstly, that HA binding to the fetuin glycoprotein facilitates NA activity (and blocking this binding reduces NA activity) and, secondly, that HA antibodies can sterically hinder NA activity by blocking the active site of the enzyme (24). These data taken together clearly demonstrate the need to use mismatched antigens containing HA not circulating in the human population, or an alternative source of antigen such as rNA or lentiviral pseudotypes.

rNA has previously been tested as an antigen in ELLA for a limited number of influenza strains [N1 from H5N1 A/Vietnam/1203/2004 (23), N1 from A/California/07/2009 and A/turkey/Turkey/01/2005 (42)]. rNA has several advantages over live virus as a source of NA for ELLA; rNA is safer to produce and handle than RG mismatch viruses without the need for high containment level facilities; rNA is easier to produce for non-influenza specialist laboratories [for example expression of tetrabrachion rNA in mammalian cells (42)] and is commercially available for some influenza strains, although this remains an expensive source of rNA.

In this study we successfully used tetrabrachion rNA against all four seasonal influenza vaccine components. Some interesting differences are observed in NI titres when testing with two different buffers, with an acidic MES buffer giving higher titres (substantially so for B/Phuket NA) than PBS buffer. MES buffer was initially selected as it has been reported that some influenza strains have impaired NA activity at neutral pH (18). B/Phuket NA appears to be pH sensitive, with higher NI titres in MES-based acidic buffer, in contrast to A/Belgium NA which does not appear to be so pH sensitive. It is not clear however, if a difference in NI titre is due to pH or perhaps the concentration of calcium within the buffer; calcium concentration is higher in MES buffer than PBS, and calcium binding is known to be important in NA activity (43–45) and NA thermostability (42, 45, 46).

Despite the differences in titre observed when using different buffers and different NA sources, the use of a calibrator reduced variation and results were comparable, with overall GCV falling from 105% to 24%. These results demonstrate that whilst rNA can be used as a source of NA in ELLA care needs to be taken in the absence of commercially available standards when comparing rNA and RG mismatched virus ELLA-NI titres.

Alternative sources of NA for ELLA-NI not investigated in this study would be detergent disrupted WT virus, or lentiviral pseudotypes expressing the NA of interest with a mismatched HA, or without HA. Lentiviral pseudotypes have been shown to give comparable titres for RG mismatched viruses for A/California/07/2009 N1 and A/Hong Kong/4801/2014 N2 (26), and it would be interesting to further test these as a source of NA using our harmonized protocol in future work.

In summary, in this study we have provided a detailed harmonized SOP for ELLA-NI, we have validated this SOP in a multi-laboratory collaborative study showing the assay had consistent precision, linearity and robustness using an N1 antigen and had influenza type specificity, including differentiation between B Yamagata and B Victoria lineages. We have shown that ELLA-NI gave consistent results when testing A and B influenza RG mismatched viruses, and additionally that rNA could be used as an alternative source of NA in the assay. We show that, in the absence of a commercially available standard, a calibrator significantly improved both inter-laboratory agreement and agreement in testing between RG mismatched viruses and rNA sources. Our results support the further development of seasonal influenza serology standards. Altogether, the validated ELLA-NI procedure and the additional specific aspects investigated are considered of great value for the harmonized and sound evaluation of immunogenicity of novel influenza vaccines and could be readily included into existing regulatory recommendations.

## Data Availability Statement

The original contributions presented in the study are included in the article/**Supplementary Material**. Further inquiries can be directed to the corresponding author.

## Ethics Statement

The studies involving human participants were reviewed and approved by Universitair Zeikenhuis Gent, Commissie vor medische ethiek (committee for medical ethics) Belgian registration number B670201733136, and the Human Biological Sample Operating Committee (HBSOC) Sanofi Pasteur. The patients/participants provided their written informed consent to participate in this study. The animal study was reviewed and approved by UK Home Office Licences and approved by NIBSC’s Animal Welfare and Ethics Review Body (AWERB). BALB/c ByJ mice were housed at Sanofi Pasteur, SA. The pooled sera were prepared from an Animal Use Protocol reviewed by the Ethics Committee #11 of Sanofi Pasteur. All experiments were conducted in accordance with the European Directive 2010/63/UE as published in the French Official Journal of February 7th, 2013.

## Author Contributions

Conceptualization and study design M-CB, HS-S, TO, DF, SG, KH, RJC, RW, EM, JPW, JW, and OE. Laboratory work HS-S, SC, LS, JW, FZ, SH, CMT, SM, SW, PA, and VL. Data analysis DF, SG, TO, and JW. Writing and Editing JW, M-CB, HS-S, SG, TO, CMT, SM, OE, and RW. All authors had full access to the data and approved the final draft of the manuscript before it was submitted by the corresponding author.

## Funding

This study was funded by the Innovative Medicines Initial Joint Undertaking (IMI JU) under grant agreement 115672, with financial contribution from the European Union Seventh Framework Programme (FP/2007-2013) and EFPIA companies’ in-kind contribution.

## Conflict of Interest

TO, DF, and SG were employed by the GSK group of companies. EM was employed by the company VisMederi srl in Italy. PA and SW were employed by the company Abbott. M-CB and SC were employed by Sanofi Pasteur.

The remaining authors declare that the research was conducted in the absence of any commercial or financial relationships that could be construed as a potential conflict of interest.

## Publisher’s Note

All claims expressed in this article are solely those of the authors and do not necessarily represent those of their affiliated organizations, or those of the publisher, the editors and the reviewers. Any product that may be evaluated in this article, or claim that may be made by its manufacturer, is not guaranteed or endorsed by the publisher.

## Supplementary Material

Supplementary Figure 1 Structure of chimeric RG influenza B viruses. The chimeric viruses containing HA of H9 and NAs from influenza B viruses were generated by reverse genetics technique using the pHW2000 plasmid as described earlier (30). The HA of these strains is a chimeric protein consisting of HA ectodomain of the H9N2 strain A/chicken/Beijing/2/97, and CT+TM (cytoplasmic tail + transmembrane region) from seasonal H1N1 strain A/Brisbane/59/2007. The NA of these viruses are also chimeric proteins containing an ectodomain (stalk and head) of the influenza B viruses (B/Brisbane/60/2008 or B/Phuket/3073/2013) and CT+TM from seasonal H1N1 strain A/Brisbane/59/2007.

Supplementary Figure 2 Intra-laboratory variation is consistent when testing multiple influenza subtypes. Three mismatched influenza viruses were tested using the FLUCOP ELLA-NI SOP. A serum panel of 16 samples was tested in three laboratories. Each laboratory carried out three independent runs. %GCV per sample is shown for Lab 2 (Green), Lab 3 (Blue) and Lab 5 (Teal) for each virus: B Bris (H9 with B/Brisbane/60/2008 NA, circles), B Phu (H9 with B/Phuket/3073/2013 NA, squares) and A Cal (H7N1 with A/California/07/2009 NA, triangles). Negative samples 12 and Ig- were excluded from analysis. %GCV is uniform across laboratory, sample and virus strain.

Supplementary Figure 3 Specificity of the ELLA-NI using monospecific ferret sera. Three mismatched influenza strains containing the neuraminidase from B/Phuket/3073/2013 (B/Phuket NA), B/Brisbane/60/2008 (B/Bris NA) and A/California/07/2009 (A/Cal HA) were tested with sera from ferrets challenged with a single strain of influenza (two individual ferrets per strain): B/Phuket/3073/2013 (B/Phuket Ferret 1 and 2); B/Brisbane/60/2008 (B/Bris Ferret 1 and 2) and A/California07/2009 (A/Cal Ferret 1 and 2). GMTs of at least two independent replicates are shown.

Supplementary Figure 4 Inter-laboratory performance before and after calibration. Three strains of influenza **(A)** H9 with B/Brisbane/60/08 NA, **(B)** H9 with B/Phuket/3073/13 NA and **(C)** H7N1 with A/California/07/09 NA, were tested using the FLUCOP ELLA-NI SOP. ELLA-NI titres are shown before (black) and after (blue) calibration using serum sample 20V02. Two pre-(V01)/post-(V02) vaccinations pairs were included in the testing (7V01/02 shaded blue and 24V01/02 shaded in green).

Supplementary Figure 5 Comparison of A/Belgium/145-MA/2009 rNA and mismatched HA/NA reverse genetics (RG) virus containing A/California/07/2009 NA in ELLA-NI and calibration using the mid-titre serum sample 20 V2. **(A)** ELLA-NI titres for A/California/07/2009 RG virus tested in three labs (A/Cal RG virus Lab 1/2/3) and rNA from A/Belgium/145-MA/2009 tested in one lab with either a DPBS buffer (A/Belgium rNA (PBS)) or an MES buffer (A/Belgium rNA (MES)). **(B)** ELLA-NI titres as in [A] after calibration with mid-titre serum sample 20 V2. **(C)** GMR of A/California/07/2009 RG virus and A/Belgium/145-MA/2009 rNA ELLA-NI titres before and after calibration. **(D)** %GCV per sample across the 5 labs/methods before and after calibration. * indicates significance using the Wilcoxon matched pairs test [P=0.0295].
