# Supplementary material for: Validation of a Harmonized Enzyme-Linked-Lectin-Assay (ELLA-NI) Based Neuraminidase Inhibition Assay Standard Operating Procedure (SOP) for Quantification of N1 Influenza Antibodies and the Use of a Calibrator to Improve the Reproducibility of the ELLA-NI With Reverse Genetics Viral and Recombinant Neuraminidase Antigens: A FLUCOP Collaborative Study
Source: Front Immunol. 2022 Jun 17;13:909297. doi: 10.3389/fimmu.2022.909297 (PMC9248865; doi:10.3389/fimmu.2022.909297)
Supplement: Supplementary file 1 [file DataSheet_1.pdf]

Enzyme-Linked Lectin Assay (ELLA) to measure Influenza-Neuraminidase-Inhibiting Antibody Titers

1 Purpose

This method is used to quantify antibody titer targeting influenza Neuraminidase in clinical serum samples pre- and post-vaccination by Enzyme-Linked Lectin Assay (ELLA).

2 Scope

This method is qualified to test serum samples against N1, N2 and B influenza neuraminidases.

3 Principle

In this method, the influenza neuraminidase (NA) enzymatic activity that cleaves terminal sialic acid moieties from highly glycosylated protein such as the fetuin is used to measure NA inhibiting antibodies in sera.

The ELLA measures the amount of penultimate galactose that becomes available after the terminal sialic acid is cleaved from the fetuin substrate (attached on a solid phase) in the presence of NA source such as mismatched virus or recombinant NA protein. The subsequent incubation with a Peroxidase-labeled peanut agglutinin (PNA), specifically recognizing galactose, followed by an incubation with an enzyme substrate-chromogen reagent causes color to develop. The intensity of the signal after addition of the substrate is dependent on the level of desialylation and thus NA activity. In the neuraminidase inhibition (NI) ELLA assay, binding of NA by specific antibodies will inhibit the enzymatic function of NA and result in a reduction of desialylation and hence, in a reduction of the signal. The color intensity is inversely proportional to the amount of inhibiting NA antibodies present in the serum. The titer of a tested serum is expressed as the reciprocal of the last dilution with an OD equal to the cut-off value corresponding to 50% end-point titer determined according to a defined formula based on virus control and the background OD. Figure 1 shows a cartoon of the ELLA principle. Appendix 10.1 gives a synopsis of the method and 10.2 a timeline for testing a new antigen/sera.

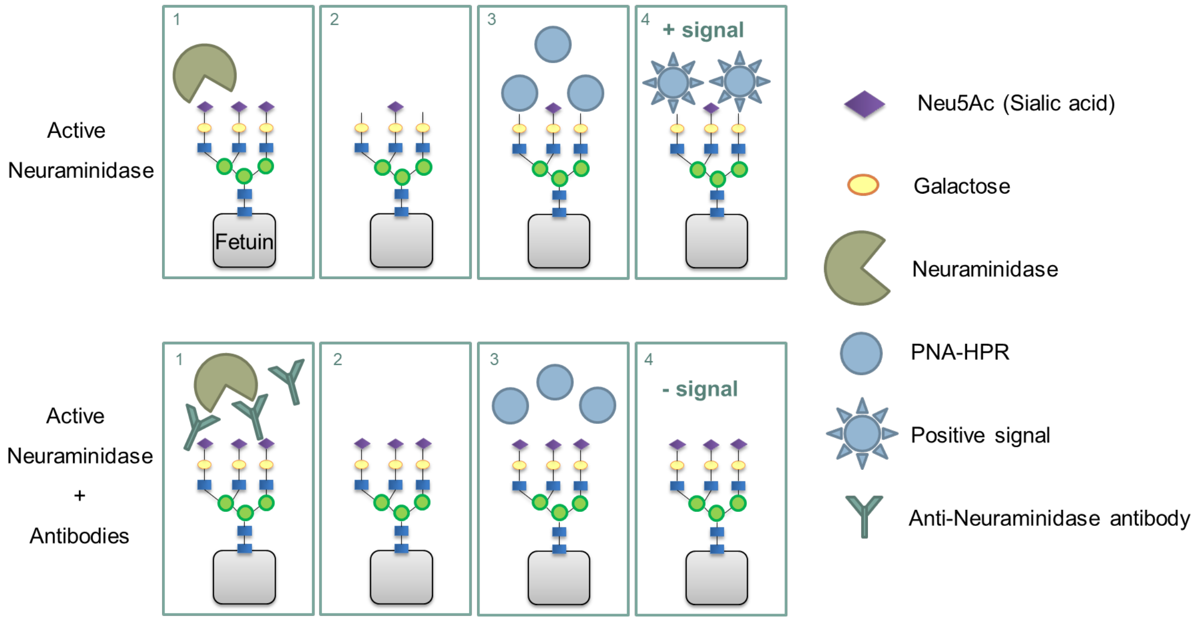

Figure 1: Enzyme-Linked-Lectin-Assay (ELLA) principle

4 Equipment and Materials

4.1 Equipment

- Class II biological safety cabinet
- Humidified incubator +37°C ± 2°C
- Water bath +56°C ± 2°C or Thermoblock
- Refrigerator +5°C ± 3°C
- Freezer ≤ 20°C and ≤ -70°C
- Plate washer (Atlas M384/Titan Stacker Biohit or equivalent)
- Vortex
- Pipette aid
- Microtiter plate reader capable of reading absorbance at 450/650 nm (Versamax Molecular Devices or equivalent)
- Timer

4.2 Materials

- Nunc-Immuno™ MicroWell™ 96 well solid plates / 96 well MicroWell™ MaxiSorp™ flat bottom plate (ThermoFisher scientific #430341 or #439454, do not substitute)
- Tubes 15 and 50mL (Falcon or equivalent)

- 1.5mL Sterile microtubes (Eppendorf or equivalent)
- Pipettes of different volumes and corresponding tips
- Serological pipettes
- Reagent reservoirs (VWM or equivalent)
- Multi-Channel Pipettes (manual or electronic) and corresponding tips (filter required in case of work with active virus)
- Aluminum foil
- Plates sealers

4.3 Reagents

- 10x coating buffer (KPL-Sera lab, cat# 5150-0014, or PBS W/O Ca and Mg Eurobio #CXXPBS01-07 or equivalent)
- Deionized H2O
- Fetuin: Sigma, cat# F3385
- Dulbecco's 1X PBS (1X DPBS) with 0.9mM CaCl2 and 0.5mM MgCl2 (Life Technologies cat# 14040-091 (500 mL) or 14040-117 (1L))
- 1 X PBS pH 7,1 without CaCl2 or MgCl2 (Eurobio or equivalent)
- 30% BSA (Sigma, cat# A8327 do not substitute)
- Tween 20 (Sigma, cat# P9416, cat#P1379 or equivalent)
- Lectin from Arachis hypogaea (peanut)-Peroxidase (Sigma, cat# L7759-1MG or equivalent)
- TMB (Europa Bioproducts Ltd, cat# MO701B (stable TMB) or equivalent
- 0.5M HCl (Roth, #6790.1) or 1M HCl (Prolabo #30024.290) or equivalent
- Antigen: mismatched viruses[1] or recombinant Neuraminidase
- Receptor Destroying Enzyme (RDE II Denka-Seiken, Tokyo Japan)

4.4 Prepared solutions

4.4.1 Coating buffer

- Dilute 10mL 10X coating buffer in 90mL deionized H2O (enough for coating 10 plates).
    - Store at +5°C ± 3°C for maximum 1 month.

[1] mismatched in regard to the anti-HA antibodies present in the tested serum samples.

4.4.2 Fetuin

4.4.2.1 Fetuin stock solution

i. Dissolve Fetuin in 1X coating buffer to make 25mg/mL solution.
ii. Store 500µL aliquots at -20°C for maximum 6 months.

4.4.2.2 Fetuin Working solution

i. Prepare immediately before coating plates.
ii. Dilute stock solution 1000-fold in 1X coating buffer (Final concentration 25µg/mL).
    a. Example: dilute 500µL Fetuin stock solution in 500mL 1X coating buffer.

4.4.3 Preparation of fetuin-coated plates

i. Dispense 100µL of fetuin working solution in each well of a Nunc-Immuno™ plate.
ii. Cover each plate with a plate sealer and store the plates for at least 18 hours +5°C ± 3°C and maximum 1 month.

4.4.4 Sample Diluent: DPBS, 1% BSA, 0.5% Tween 20

i. In 96.2mL 1X DPBS
    a. Add 3.3mL 30% BSA
    b. Add 0.5mL of Tween 20
    c. Store at +5°C ± 3°C for 1 month

4.4.5 Conjugate Diluent: DPBS, 1% BSA

i. In 96.7mL DPBS
    a. Add 3.3mL 30% BSA
    b. Store at +5°C ± 3°C for 1 month

4.4.6 PNA-HRPO: Lectin from Arachis hypogaea (peanut)-Peroxidase

i. Dissolve 1mg in 1mL of conjugate diluent.
    a. Store 100µL aliquots at -20°C for maximum 6 months.
ii. Immediately before use, prepare the appropriate dilution of PNA-HRPO in **conjugate diluent** according to the number of plates to test.

For example: for a dilution of 1000-fold for 10 plates:

- Dilute 11µL of PNA-HRPO in 11mL of **Conjugate diluent**.

iii. When using a new PNA lot, determine the dilution that results:
    a. in a maximal OD of 0.1 for the background (no antigen, no serum).
    b. in a maximal OD leading to a plateau for the antigen control.
        i. A dilution of 1:1000 is usually recommended.

Note: The use of the same batch of PNA is required to for a defined study

4.4.7 Wash buffer: 1X PBS pH7.1, 0.05% Tween 20 (PBS-T)

i. Dilute 0.5mL of Tween 20 in 1L 1X PBS
    a. Store at RT or at +5°C ± 3°C for up to 3 months.

4.4.8 Substrate Solution (TMB)

Ready to use solution, stored at +5°C ± 3°C.

4.4.9 Stop Solution (HCl)

Ready to use solution, stored at RT in a chemical cabinet. 0.5M HCl or 1M HCl do not have an impact on the results. Both molarities can be used to stop the assay.

5 Safety

When ELLA is performed using infectious influenza virus as source of NA, all steps should be performed under class II biological safety cabinet.

6 Procedure

6.1 Sample preparation

i. Test and control sera should be heat-inactivated at 56°C for 45-60 min.
ii. Some sera contain non-specific inhibitors of NA. These sera will give a false positive signal in ELLA when tested with heterologous antigen. In the absence of screening samples for non-specific inhibition of NA, we recommend treating sera with Receptor Destroying Enzyme (RDE) to remove non-specific inhibitors prior to testing (detailed protocol given in section 9.1).
iii. Store sera (before or after treatment) at -20 to -70°C
    a. If samples are to be tested repeatedly, prepare several aliquots to avoid repeated freeze/thawing cycle.

6.2 Antigen titration

The antigen source can be either a mismatched virulent influenza virus (clarified allantoïc fluid or cell culture fluid), a mismatched inactivated influenza virus (by β-propriolactone) or a recombinant NA (rNA).

Before proceeding with the serum sample titration, the amount of antigen to be used in the ELLA should be determined in at least 3 independent titrations (performed on different days).

The titer of the antigen for each titration is determined by the value of the reciprocal dilution giving 90% of the maximal OD signal. The final titer of the antigen is the average of at least 3 titer determinations (see 7.1).

Note: All dilution steps can be directly performed in the fetuin-coated plate.

6.2.1 Antigen preparation

i. Thaw an aliquot of antigen and keep on ice or at +5°C ± 3°C.
ii. Prepare at least two different antigen starting dilution: 1/10 and 1/100
    a. 1/10 dilution: 4.5mL of sample diluent + 500µL of antigen stock solution.
    b. 1/100 dilution: 4.95mL of sample diluent + 50µL of antigen stock solution.
iii. Store all preparation on ice or at +5°C ± 3°C

6.2.2 Fetuin-coated plate preparation

i. Take fetuin-coated plate out from cold storage (+5°C ± 3°C). Aspirate fetuin solution from each well.
ii. Wash fetuin-coated plate 3 times with at least 150µL of wash buffer with an electronic multichannel pipette, with an automatic dispenser or with a microplate washer.
iii. Blot each plate onto absorbent paper towel immediately before use to remove excess wash buffer

6.2.3 Titration and Incubation of antigen

iv. Aliquot 50µL sample diluent into all wells of column 2-12 of the fetuin-coated plate.
v. Dispense 100µL of each antigen dilution directly in the well of column 1.
vi. Make 2-fold serial dilutions of antigen in sample diluent, by transferring 50µL from one well to the next with same tips.
    a. Discard the 50µL samples left from column 12.
vii. Add 50µL of sample diluent to row H as negative control.
viii. Add 50µL of sample diluent to each well in column 1-12 of fetuin-coated plate (except row H).
ix. Final volume in each well should be 100µL.
x. Cover with plate sealer
xi. Place plate(s) in a humidified incubator at 37°C ± 1°C and incubate for 20h ± 1h.

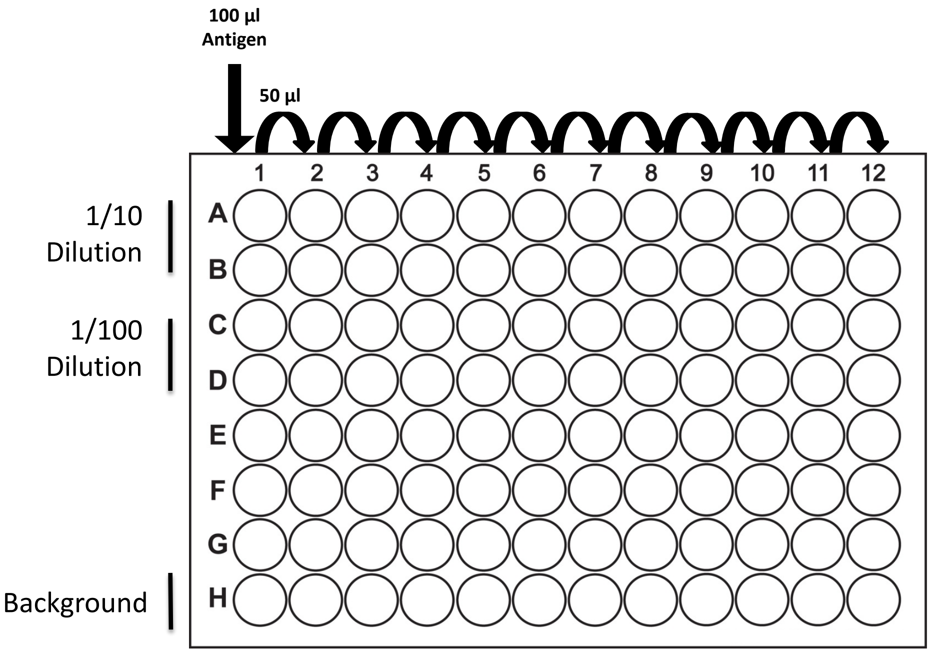

6.2.4 Assay development

i. Prepare PNA-HRPO solution as described in 4.4.6.
   a. Note: always prepare an extra volume of solution (each plate requires 10mL of solution).
ii. Transfer plates from the incubator to the pipetting station and carefully remove all plate seals to avoid spills.
iii. Wash all plates 6 times with at least 150µL of wash buffer with an electronic multichannel pipette, with an automatic dispenser or with a microplate washer. Invert each plate and blot onto absorbent paper towel to ensure that all liquid is removed from the wells.
iv. Add 100µL/well PNA-HRPO solution (at the dilution determined previously, see 4.4.6) to all test wells on all plates.
v. Incubate the plates for 120 min ± 15 min at RT in the dark.
vi. Wash the test plates 3 times to remove the PNA-HRPO with at least 150µL of wash buffer with an electronic multichannel pipette, with an automatic dispenser or with a microplate washer.
vii. Invert each plate and blot onto absorbent paper towel.
viii. Add 100µL of TMB to each well on all plates (TMB should be at 4°C)
ix. Incubate the plates for 20 min ± 5 min at room temperature in the dark.
x. Stop the reaction by adding 100µL/well of 0.5M HCl or 1M HCl.
xi. Read the Optical Density (OD) of all the test plates at 450/650 nm.
xii. Save and export all data files.

6.3 Back-titration

Antigen back-titration should be performed at least once before the first serum titration experiment.

Then, the back-titration should be performed on each plate of serum titration.

6.3.1 Antigen preparation

i. Thaw an aliquot of antigen and keep on ice or at +5°C ± 3°C.
ii. Prepare a 4-fold lower antigen dilution in 10mL sample diluent. 14mL reflects the volume required for 10 plates serum titration.
    a. Example: 90% max = 1/652, 4-fold lower dilution = 1/163 (antigen is 4X concentrated)
        i. Dilute 86µL antigen in 13.914mL sample diluent.
    b. Store dilution on ice or at +5°C ± 3°C

6.3.2 Fetuin-coated plate preparation

i. Aspirate remaining coating buffer from each well.
ii. Wash fetuin-coated plate 3 times with at least 150µL of wash buffer with an electronic multichannel pipette, with an automatic dispenser or with a microplate washer.
iii. Blot each plate onto absorbent paper towel immediately before use to remove excess wash buffer.

6.3.3 Titration and Incubation of antigen

i. Aliquot 50µL sample diluent into wells of column 2-12 of the fetuin-coated plate.
ii. Dispense 100µL of each antigen dilution directly in the well of column 1.
iii. Make 2-fold serial dilutions of antigen in sample diluent, by transferring 50µL from one well to the next with the same tips.
    a. Discard the 50µL samples left from column 12.
iv. Add 50µL of sample diluent to row H as negative control.
v. Add 50µL of sample diluent to each well in column 1-12 of fetuin-coated plate (except row H).
vi. Final volume in each well should be 100µL.
vii. Cover with plate sealer
viii. Place plate(s) in a humidified incubator at 37°C ± 1°C and incubate for 20h ± 1h.

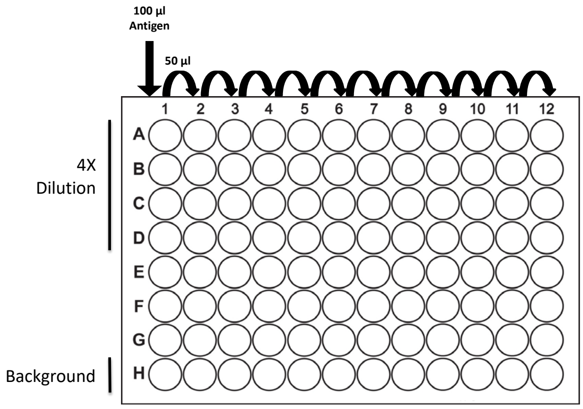

or

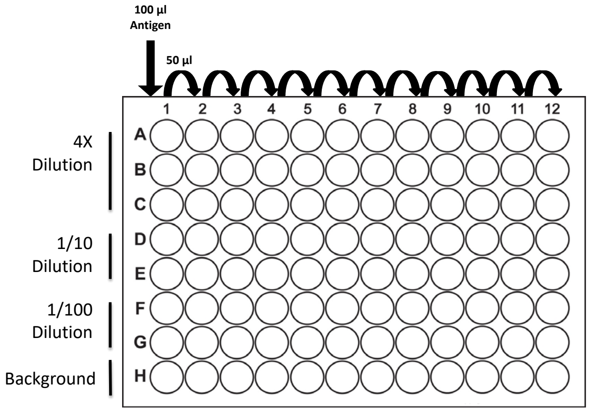

6.3.4 Assay development

i. Prepare PNA-HRPO solution as explained in 4.4.6.
   a. Note: always prepare an extra volume of solution (each plate requires 10mL of solution).
ii. Transfer plates from the incubator to the pipetting station and carefully remove all plate seals to avoid spills.
iii. Wash all plates 6 times with at least 150µL of wash buffer with an electronic multichannel pipette, with an automatic dispenser or with a microplate washer.
iv. Invert each plate and pat to ensure all liquid has been removed from wells
v. Add 100µL/well PNA-HRPO solution (at the dilution determined previously, see 4.4.6) to all test wells on all plates.
vi. Incubate the plates for 120 min ± 15 min at RT in the dark.
vii. Wash the test plates 3 times to remove the PNA- HRPO with at least 150µL of wash buffer with an electronic multichannel pipette, with an automatic dispenser or with a microplate washer.
viii. Invert each plate and blot onto absorbent paper towel.
ix. Add 100µL of TMB to each well on all plates (TMB should be at 4°C)
x. Incubate the plates for 20 min ± 5 min at room temperature in the dark.
xi. Stop the reaction by adding 100µL/well of 0.5M HCl or 1M HCl.
xii. Read the Optical Density (OD) of all the test plates at 450/650 nm.
xiii. Save and export all data files.

6.4 Serum titration

Once the antigen titer corresponding to 90% of the maximal activity is determined, the test and control sera samples are ready to be titrated.

During the serum titration a maximum of **10 plates** can be handled in one run.

6.4.1 Serum preparation

i. Thaw a vial of each control and test sera samples and keep on ice or at +5°C ± 3°C.
ii. Prepare 1/10 dilution of each sera.
  a. 1/10 dilution:
    i. If directly diluted in the plate: 90µL of sample diluent + 10µL of inactivated serum
    ii. If prepared in tube: 135µL of sample diluent + 15µL of inactivated serum
    iii. If directly diluted in the plate with RDE treated sera: 50µL of sample diluent + 50µL of RDE treated sera (see 9.1).
  b. Store all preparation on ice or at +5°C ± 3°C

6.4.2 Antigen preparation

Thaw an aliquot of antigen and keep on ice or at +5°C ± 3°C.

6.4.2.1 Preparation of 4X antigen dilution

Prepare a 4-fold lower antigen dilution in 10mL sample diluent. 14mL reflects the volume required for 10 plates serum titration. This preparation is used for the back-titration and for the preparation of the 1X antigen dilution.

i. Example: 90% max = 1/ 652, 4-fold lower dilution = 1/163 (antigen is 4X concentrated)
  a. Dilute 86µL antigen in 13.914mL sample diluent.
ii. Store dilution on ice or at +5°C ± 3°C

6.4.2.2 Preparation of 1X Antigen dilution

Prepare a 1X antigen dilution from the 4X antigen dilution in 50mL sample diluent. 50mL reflects the volume required for 10 plates serum titration.

i. Dilute 12.5mL of 4X antigen dilution in 37.5mL sample diluent.
ii. Store dilution on ice or at +5°C ± 3°C

6.4.3 Fetuin-coated plate preparation

i. Aspirate remaining coating buffer from each well.
ii. Wash fetuin-coated plate 3 times with at least 150µL of wash buffer with an electronic multichannel pipette, with an automatic dispenser or with a microplate washer.
iii. Blot each plate onto absorbent paper towel immediately before use to remove excess wash buffer

6.4.4 Titration of serum and Incubation with antigen

i. Aliquot 50µL sample diluent into wells of column 2-12 of the fetuin-coated plate and well H1.
ii. Dispense in the well of column 1 (well A1 to F1) either:
   - 100µL of a 1/10 serum dilution
   - 10µL of neat serum containing 90µL of sample diluent
iii. Dispense 100µL of 4X Antigen dilution in the well G1
iv. Dispense 50µL sample diluent into wells H7 to H12
v. Make 2-fold serial dilutions of the sera and back-titration antigen in sample diluent (rows A to G), by transferring 50µL from one well to the next with the same tips.
   a. Discard the 50µL samples left from column 12.
vi. Add 50µL of 1X antigen dilution to row A to F and well H1 to H6.
vii. Add 50µL of sample diluent to each well in row G.
viii. Final volume in each well should be 100µL.
ix. Cover with plate sealer
x. Place plate(s) in a humidified incubator at 37°C ± 1°C and incubate for 20 h ± 1h.

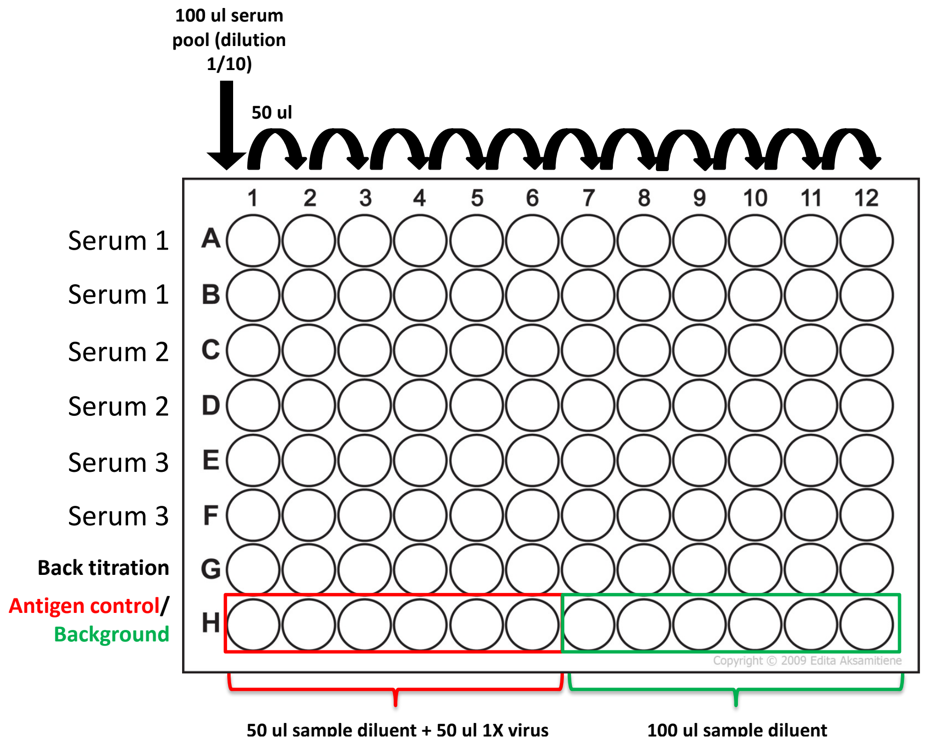

6.4.5 Assay development

i. Prepare PNA-HRPO solution as explained in 4.4.6.
   a. Note: always prepare an extra volume of solution (each plate requires 10 mL of solution).
ii. Transfer plates from the incubator to the pipetting station and carefully remove all plate seals to avoid spills.

iii. Wash all plates 6 times with at least 150µL of wash buffer with an electronic multichannel pipette, with an automatic dispenser or with a microplate washer. Invert each plate and pat to ensure all liquid has been removed from wells
iv. Add 100µL/well PNA-HRPO solution (at the dilution determined previously, see 4.4.6) to all test wells on all plates.
v. Incubate the plates for 120 min ± 15 min at RT in the dark.
vi. Wash the test plates 3 times to remove the PNA- HRPO with at least 150µL of wash buffer with an electronic multichannel pipette, with an automatic dispenser or with a microplate washer.
vii. Invert each plate and blot onto absorbent paper towel.
viii. Add 100µL of TMB to each well on all plates (TMB should be at 4°C)
ix. Incubate the plates for 20 min ± 5 min at room temperature in the dark.
x. Stop the reaction by adding 100µL/well of 0.5M HCl or 1M HCl.

- Read the Optical Density (OD) of all the test plates at 450/650 nm.
- Save and export all data files.

7 Titer Calculation

7.1 Antigen titer

The antigen titer corresponds to the reverse dilution corresponding to 90% of the neuraminidase activity using the sigmoidal dose-response (4 parameters function).

The calculation can be performed using either GraphPAD PRISM, SoftMax Pro Software or any other statistical software.

The Flucop Excel Calculation sheet can also be used.

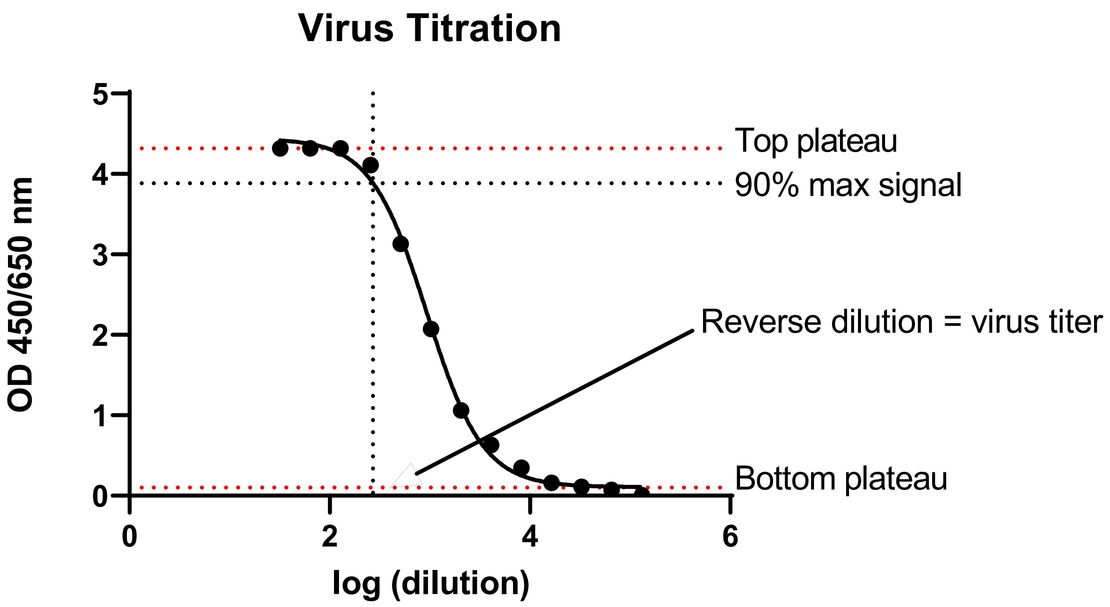

The final titer of the antigen is the geometric mean of at least 3 independent antigen titrations. Note: titration values used to calculate the geometric mean should be within a 4-fold range from lowest to highest.

For example:

- if the antigen titration leads to:
  - 640
  - 320
  - 160
    - The 4-fold range between 640 and 160 is kept; all titers can be used to calculate the geometric mean.
- If the antigen titration leads to:
  - 1280
  - 320
  - 160
    - The 4-fold range between 1280 and 160 is not kept, the outlier should be removed from the calculation and a supplementary titer should be performed.

Same principle is used to calculate the back-titration corresponding to the 4X antigen dilution.

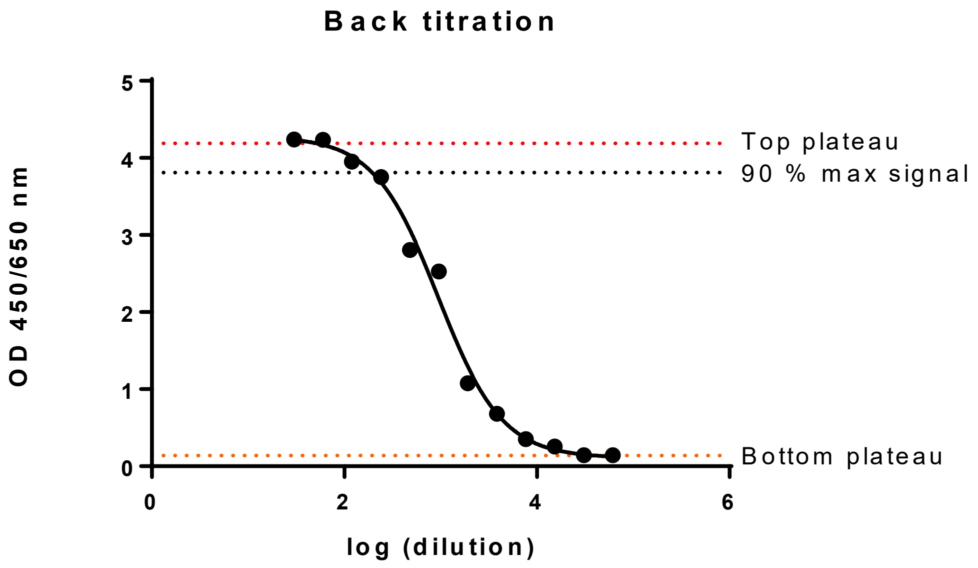

7.2 Serum titer

The serum titer corresponds to the reverse dilution **equal** to the cut-off.

The cut-off can be determined via the following formula:

$$\frac{Mean\ OD\ of\ virus\ control\ cells - Mean\ OD\ of\ background\ wells}{2} + mean\ OD\ background\ wells = cut-off\ (corresponding\ to\ 50\%\ end\ point\ titer)$$

The serum titer is calculated using the sigmoidal dose-response (4 parameters function) with either GraphPAD PRISM, SoftMax Pro Software or any other statistical software titer.

The Flucop Excel Calculation sheet can also be used.

If the cut-off value was not reached at any dilution, the titer is less than the first dilution tested, *e.g.*, <10 when 1:10 is the first dilution tested, an arbitrary titer of 5 is then assigned.

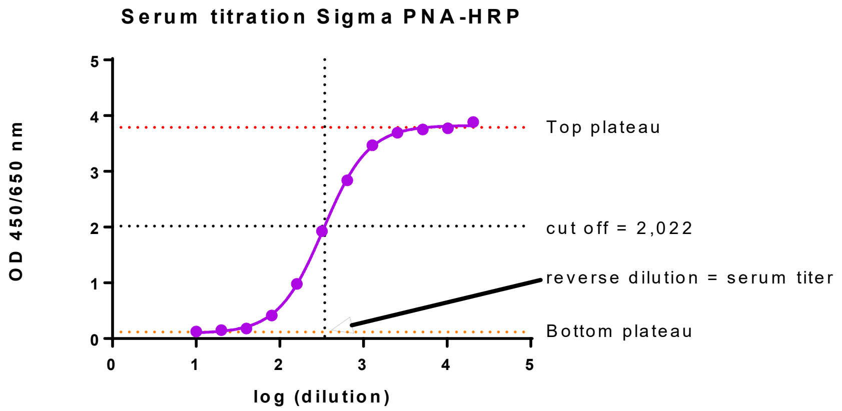

8 Acceptance Criteria

8.1 Assay acceptance criteria

The plate is valid if:

i. Background (no antigen, no serum) value of each well is below OD 0.1.
   a. Outliers may be removed to a maximum of 2 out of the 6 wells per plate.
ii. Antigen control (antigen, no serum).
   a. Outliers may be removed to a maximum of 2 out of the 6 wells per plate.

iii. Back-titration 4X antigen dilution (present on each plate) should not differ by more than 2-fold from the target antigen titer (determined in 6.2).

8.2 Retesting criteria

Samples of one plate will be retested if one of the acceptance criteria listed above is not met.

9 Recommendations

9.1 Serum treatment with Receptor Destroying Enzyme (RDE)

Some sera contain non-specific inhibitors of NA. These sera will give a false positive signal in ELLA when tested with heterologous antigen. In the absence of screening samples for non-specific inhibition of NA, we recommend treating **all** sera with Receptor Destroying Enzyme (RDE) to remove non-specific inhibitors prior to testing.

RDE should be diluted 1 in 10. Four parts diluted RDE are added to 1 part sera and incubated at 37°C overnight. **RDE must be heat inactivated for 8h at 56°C** (a longer heat inactivation is required to completely remove RDE activity, as active RDE will produce a signal in ELLA).

Caution: As sera are now diluted 1 in 5, the volume required for serum titration would be 50µL sera and 50µL sample buffer.

9.2 Hemagglutinin Inhibition (HAI) test

It is recommended to perform an HAI test on serum samples with the same mismatched virus subsequently used when proceeding with the ELLA test. This step can already inform on the presence of HA antibodies potentially recognizing the HA protein of the mismatched virus, which could subsequently affect the NA-antibodies titer.

9.3 Control serum

Recommendation: Inclusion of at least one positive control serum which will be deposited in one of the different plates of each experiment. When the limits of a control chart are established, the titer of that serum will have to be included in those limits.

9.4 Plasma samples

Recommendation: This SOP is performed only with serum samples, however plasma samples might also be selected but previously screened for the presence of interfering factors (for example chelating agents) prior to use in ELLA.

9.5 Antigen or serum titration

When performing the antigen titration using a mismatched virus (active or inactivated), the obtained titer should be within the range 100-1000.

For titration performed with a recombinant protein, the range might be in a much higher range as for a virus.

9.5.1 Curve analysis of antigen or serum titration

During an antigen titration or serum titration one aberrant point can be deleted from the titration curve

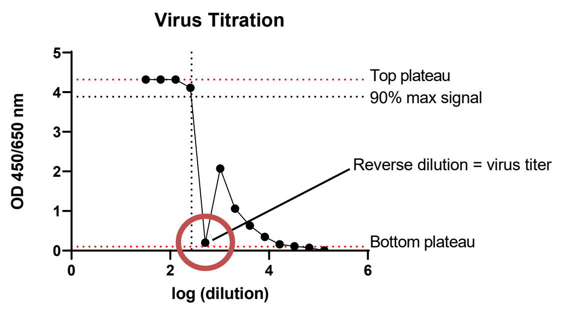

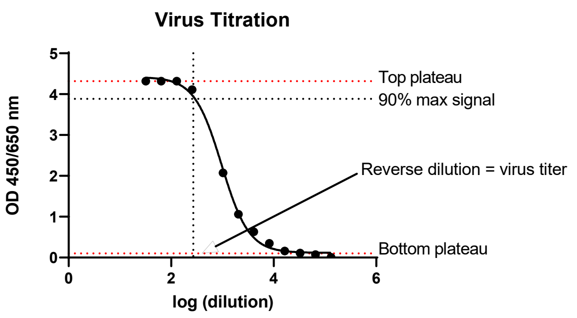

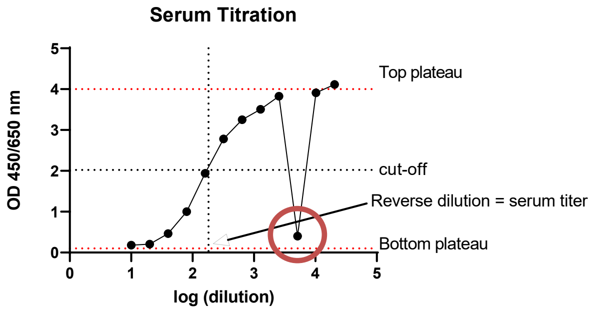

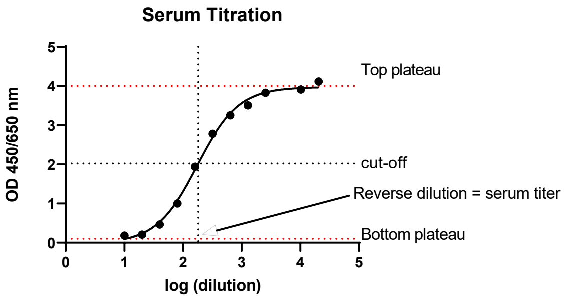

10 Appendix

10.1 Synopsis of the ELLA Method

ELLA<br>Summary of the Operating Method		
Step	*Reagents – Dilutions*	*Buffers*
Pr-Coating plates	**100 µL per well of Fetuin at 25µg/mL**<br>Incubation : 18hours to 1 month at +5°C ± 3°C	**PBS w/o Ca &Mg**
**Empty the plates / 3 Washes**		**Wash buffer**
Serum titration<br><br>Addition of antigen	**100 µL per well (50µl serum dilution usually at 1:10**<br>**+ 50µL of virus at the appropriate dilution corresponding to 90% of the maximum OD value)**<br>Incubation : 20hours at +37°C ± 2°C	**Sample diluent**
**6 Washes**		**Wash buffer**
Addition of HRP PNA	**100 µL per well of usually 1:1000 dilution**<br>Incubation : 2 h ± 15 minutes at Room Temperature	**Conjugate diluent**
**3 Washes**		**Wash buffer**
Detection	TMB : 100 µL p **per well**<br>Incubation : 20 minutes ± 5 minutes in the dark at room temperature	**N/A**
Stop	**HCl 0.5 or1N : 100 µL per well**	
**Reading on Microtiter plate reader (450-650 nm)**		

10.2 Timeline for testing of new antigen/sera

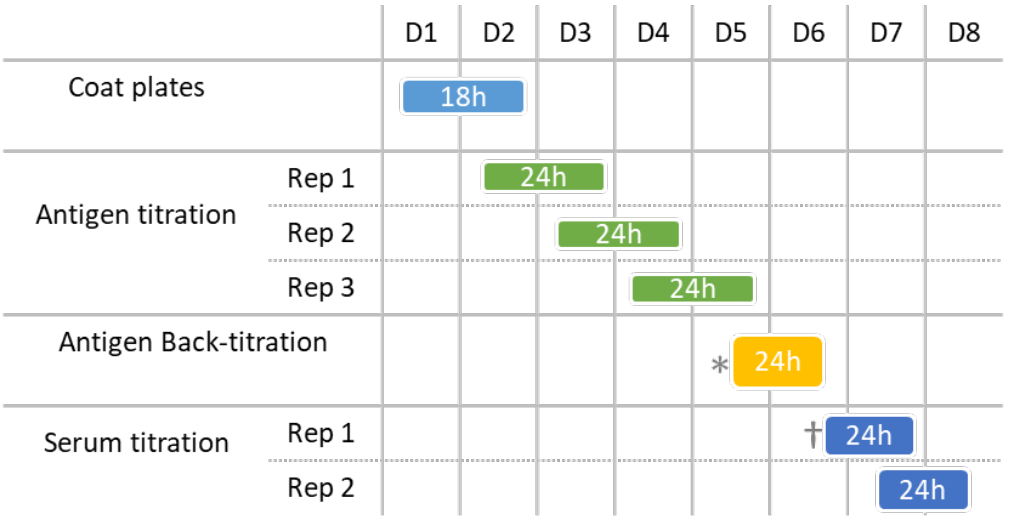

* Requires average of three titration results

† Back-titration should be within 2-fold of average antigen titration
