# Supplementary figures and images for: Validation of a Harmonized Enzyme-Linked-Lectin-Assay (ELLA-NI) Based Neuraminidase Inhibition Assay Standard Operating Procedure (SOP) for Quantification of N1 Influenza Antibodies and the Use of a Calibrator to Improve the Reproducibility of the ELLA-NI With Reverse Genetics Viral and Recombinant Neuraminidase Antigens: A FLUCOP Collaborative Study
Source: Front Immunol. 2022 Jun 17;13:909297. doi: 10.3389/fimmu.2022.909297 (PMC9248865; doi:10.3389/fimmu.2022.909297)

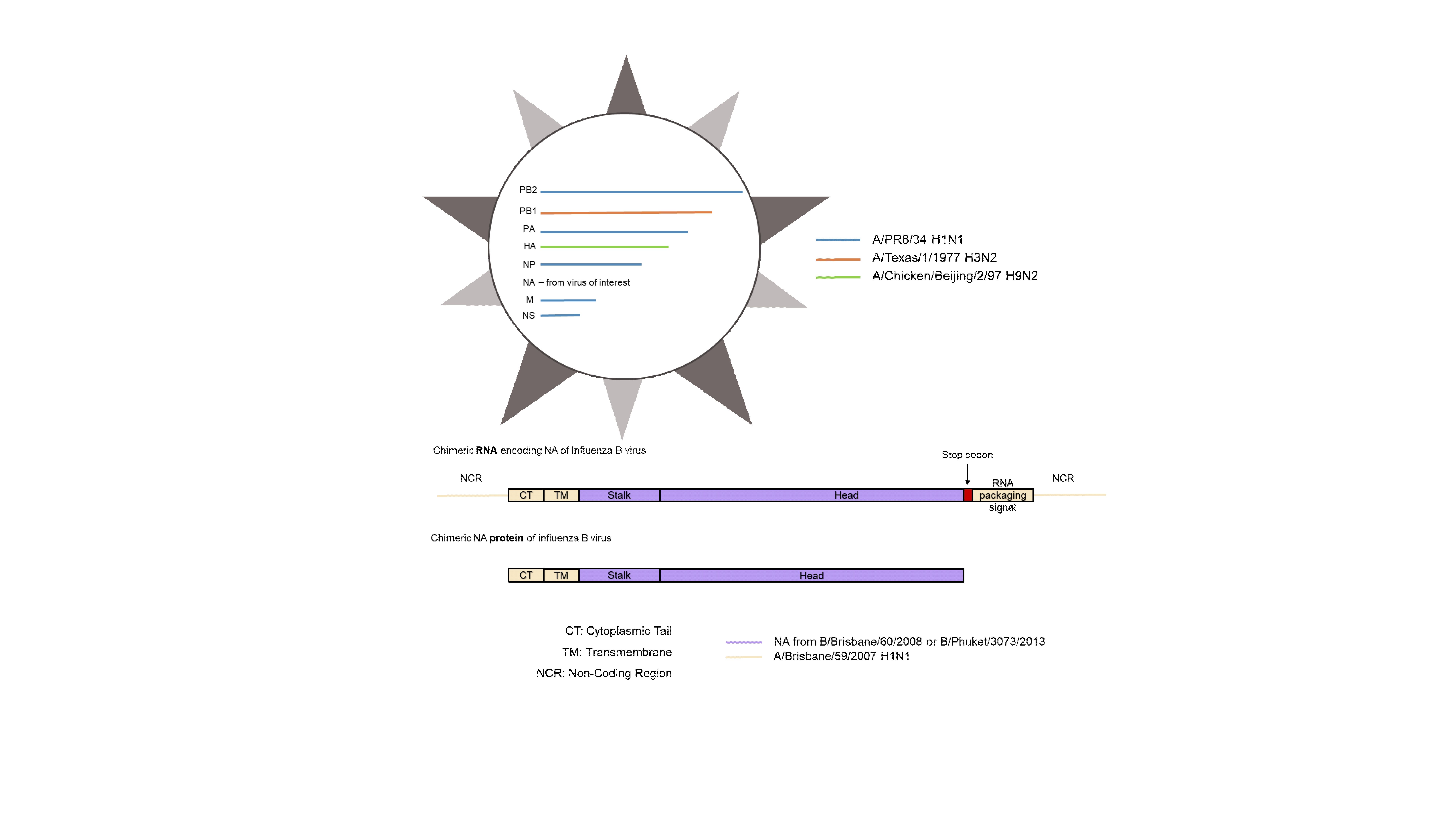

Supplement: Supplementary Figure 1 — Structure of chimeric RG influenza B viruses. The chimeric viruses containing HA of H9 and NAs from influenza B viruses were generated by reverse genetics technique using the pHW2000 plasmid as described earlier (30). The HA of these strains is a chimeric protein consisting of HA ectodomain of the H9N2 strain A/chicken/Beijing/2/97, and CT+TM (cytoplasmic tail + transmembrane region) from seasonal H1N1 strain A/Brisbane/59/2007. The NA of these viruses are also chimeric proteins containing an ectodomain (stalk and head) of the influenza B viruses (B/Brisbane/60/2008 or B/Phuket/3073/2013) and CT+TM from seasonal H1N1 strain A/Brisbane/59/2007. [file Image_1.jpeg]

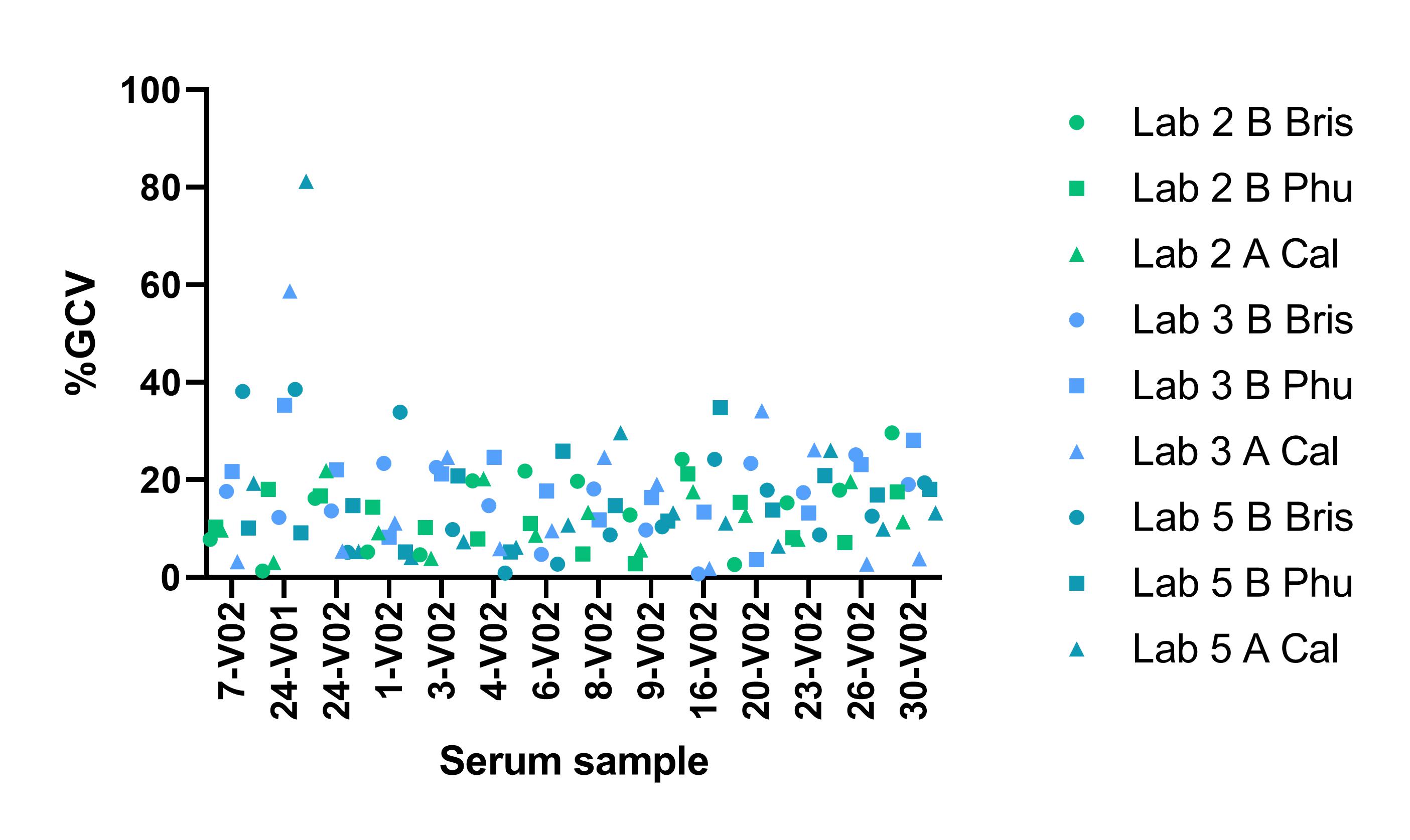

Supplement: Supplementary Figure 2 — Intra-laboratory variation is consistent when testing multiple influenza subtypes. Three mismatched influenza viruses were tested using the FLUCOP ELLA-NI SOP. A serum panel of 16 samples was tested in three laboratories. Each laboratory carried out three independent runs. %GCV per sample is shown for Lab 2 (Green), Lab 3 (Blue) and Lab 5 (Teal) for each virus: B Bris (H9 with B/Brisbane/60/2008 NA, circles), B Phu (H9 with B/Phuket/3073/2013 NA, squares) and A Cal (H7N1 with A/California/07/2009 NA, triangles). Negative samples 12 and Ig- were excluded from analysis. %GCV is uniform across laboratory, sample and virus strain. [file Image_2.jpeg]

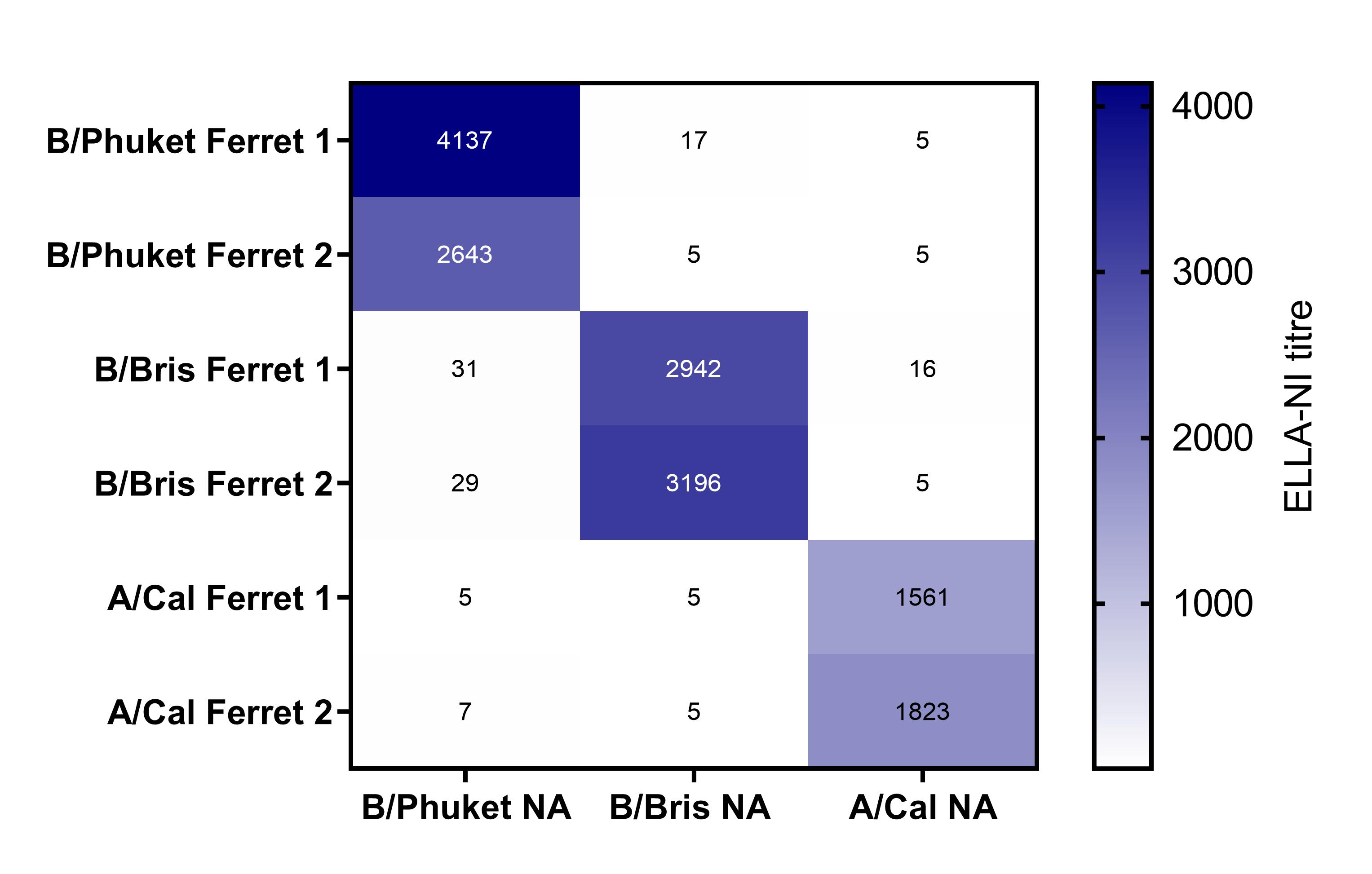

Supplement: Supplementary Figure 3 — Specificity of the ELLA-NI using monospecific ferret sera. Three mismatched influenza strains containing the neuraminidase from B/Phuket/3073/2013 (B/Phuket NA), B/Brisbane/60/2008 (B/Bris NA) and A/California/07/2009 (A/Cal HA) were tested with sera from ferrets challenged with a single strain of influenza (two individual ferrets per strain): B/Phuket/3073/2013 (B/Phuket Ferret 1 and 2); B/Brisbane/60/2008 (B/Bris Ferret 1 and 2) and A/California07/2009 (A/Cal Ferret 1 and 2). GMTs of at least two independent replicates are shown. [file Image_3.jpeg]

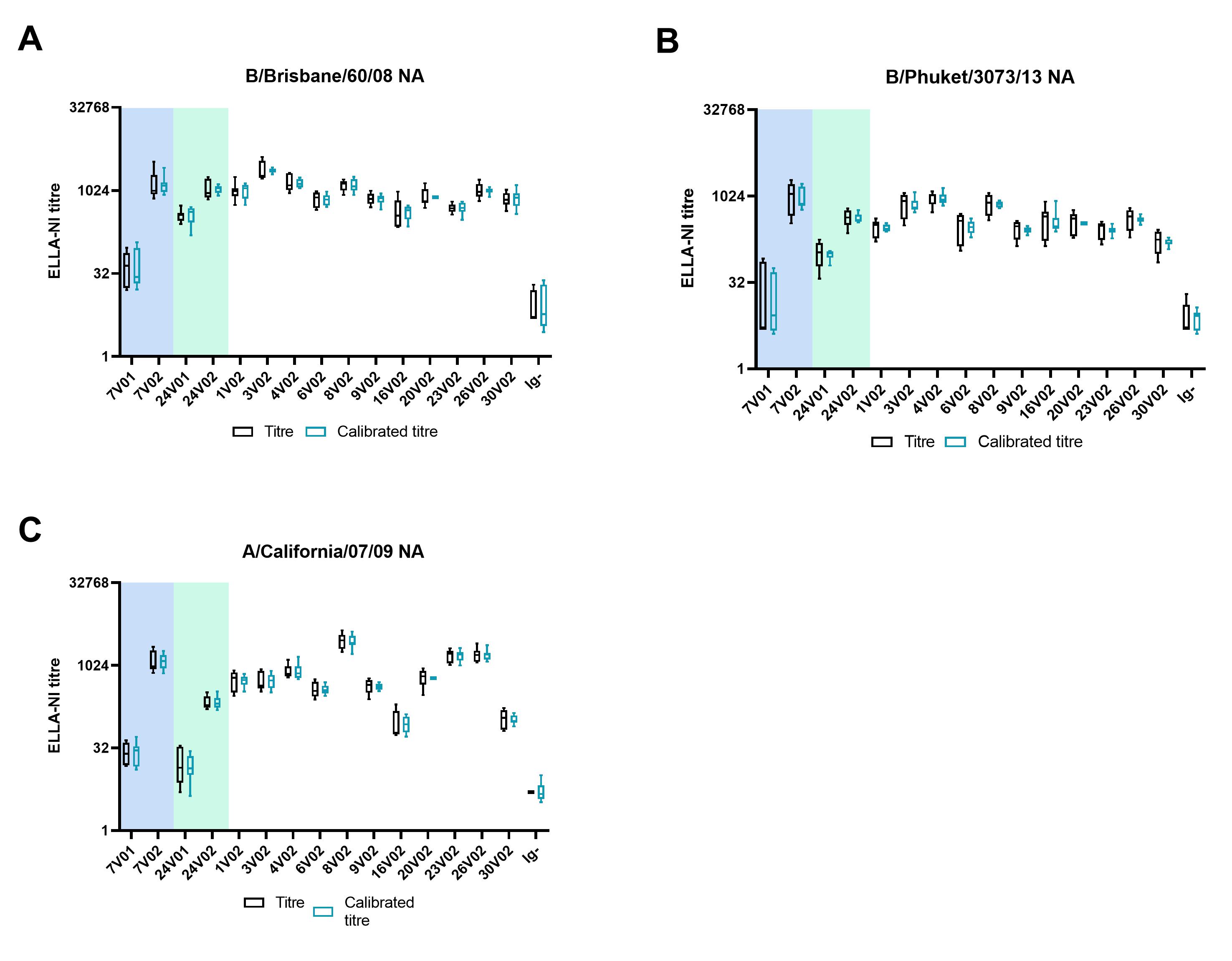

Supplement: Supplementary Figure 4 — Inter-laboratory performance before and after calibration. Three strains of influenza (A) H9 with B/Brisbane/60/08 NA, (B) H9 with B/Phuket/3073/13 NA and (C) H7N1 with A/California/07/09 NA, were tested using the FLUCOP ELLA-NI SOP. ELLA-NI titres are shown before (black) and after (blue) calibration using serum sample 20V02. Two pre-(V01)/post-(V02) vaccinations pairs were included in the testing (7V01/02 shaded blue and 24V01/02 shaded in green). [file Image_4.jpeg]

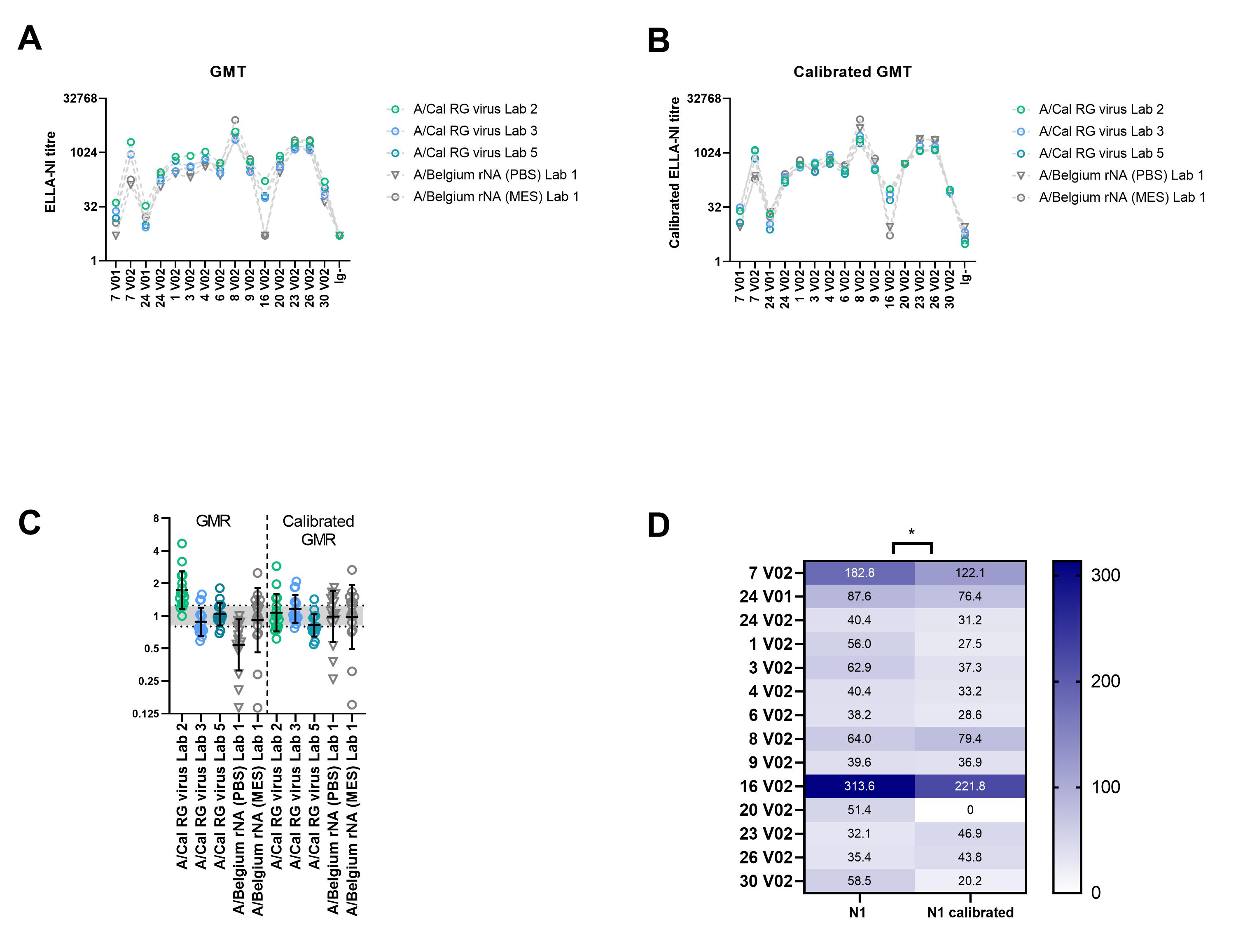

Supplement: Supplementary Figure 5 — Comparison of A/Belgium/145-MA/2009 rNA and mismatched HA/NA reverse genetics (RG) virus containing A/California/07/2009 NA in ELLA-NI and calibration using the mid-titre serum sample 20 V2. (A) ELLA-NI titres for A/California/07/2009 RG virus tested in three labs (A/Cal RG virus Lab 1/2/3) and rNA from A/Belgium/145-MA/2009 tested in one lab with either a DPBS buffer (A/Belgium rNA (PBS)) or an MES buffer (A/Belgium rNA (MES)). (B) ELLA-NI titres as in [A] after calibration with mid-titre serum sample 20 V2. (C) GMR of A/California/07/2009 RG virus and A/Belgium/145-MA/2009 rNA ELLA-NI titres before and after calibration. (D) %GCV per sample across the 5 labs/methods before and after calibration. * indicates significance using the Wilcoxon matched pairs test [P=0.0295]. [file Image_5.jpeg]
