# Supplementary material for: Validation of a Harmonized Enzyme-Linked-Lectin-Assay (ELLA-NI) Based Neuraminidase Inhibition Assay Standard Operating Procedure (SOP) for Quantification of N1 Influenza Antibodies and the Use of a Calibrator to Improve the Reproducibility of the ELLA-NI With Reverse Genetics Viral and Recombinant Neuraminidase Antigens: A FLUCOP Collaborative Study
Source: Front Immunol. 2022 Jun 17;13:909297. doi: 10.3389/fimmu.2022.909297 (PMC9248865; doi:10.3389/fimmu.2022.909297)
Supplement: Supplementary file 7 [file Table_1.pdf]

			ANOVA estimate Series 1			ANOVA estimate Series 2		
**Lab**	**Sample**	**Number of valid results**	**GMT**	**Rep CV (%)**	**IP CV (%)**	**GMT**	**Rep CV (%)**	**IP CV (%)**
1	1	16	295,0	10,4	33,7	291,9	17,3	39,0
1	2	16	159,3	7,8	28,9	169,8	14,2	29,3
1	3	16	253,3	5,4	22,3	269,2	13,4	35,1
1	4	16	30,3	17,0	34,4	27,1	6,5	26,3
1	5	16	231,1	8,0	32,4	222,5	12,1	31,9
1	6	16	424,4	8,6	35,3	381,8	7,8	29,3
1	7	16	80,8	9,2	25,0	76,5	9,2	20,7
1	8	16	2563,3	13,6	35,6	2671,7	20,9	36,2
1	9	16	4855,5	8,8	34,2	5061,7	28,7	54,2
2	1	16	172,3	9,6	13,5	134,6	26,1	30,6
2	2	16	74,9	12,2	34,0	60,4	15,4	31,0
2	3	16	132,8	19,1	32,1	104,7	11,9	19,7
2	5	16	101,1	19,8	30,2	75,6	18,8	38,4
2	6	16	183,1	9,3	23,7	151,1	17,3	31,9
2	7	16	32,0	14,3	14,8	30,4	7,7	29,4
2	8	16	981,8	17,9	24,2	887,5	3,3	13,3
2	9	16	2010,9	13,3	17,1	1657,7	11,2	20,6
3	1	16	429,7	7,4	18,0	479,0	6,7	17,9
3	2	16	202,4	7,9	22,9	234,5	5,8	15,2
3	3	16	934,6	10,8	23,4	1032,4	13,7	21,8
3	4	16	63,8	11,9	22,1	64,8	16,6	18,7
3	5	16	361,8	7,3	17,8	342,8	7,0	8,7
3	6	16	655,8	8,6	17,1	575,8	12,2	17,0
3	7	16	134,6	15,7	20,6	129,1	11,5	13,3
3	8	16	3630,6	15,1	18,2	3794,3	6,6	10,5
3	9	16	6804,6	7,1	21,8	7555,7	10,0	12,3
4	1	16	577,2	19,0	60,2	616,6	42,7	56,6
4	2	16	260,7	14,6	41,2	278,9	10,9	25,7
4	3	16	439,0	11,3	32,5	440,8	6,9	35,9
4	4	16	32,8	28,2	28,2	40,7	18,4	19,1
4	5	16	380,4	9,7	26,7	400,9	7,4	28,6
4	6	16	619,2	18,7	28,1	634,6	13,3	30,3
4	7	16	140,5	18,4	18,4	110,1	21,9	33,8
4	8	16	4232,7	19,5	26,7	4023,0	15,8	37,8
4	9	16	6473,9	26,5	35,5	7023,5	20,6	34,0
5	1	8	566,2	10,4	20,2	540,6	3,1	14,7
5	2	8	215,1	9,7	15,7	222,3	8,5	15,1
5	3	8	341,5	13,9	19,0	346,5	2,3	13,3
5	4	8	52,3	9,4	13,1	54,9	5,2	10,0
5	5	8	339,8	7,0	22,2	359,9	12,5	19,3
5	6	8	563,7	8,0	14,2	586,3	5,3	13,8
5	7	8	103,8	7,2	8,1	99,8	13,1	21,0
5	8	8	3214,7	11,3	27,0	3559,7	5,4	13,8
5	9	8	6720,0	8,5	28,1	6901,9	15,0	20,2
6	1	16	209,4	5,2	5,2	209,4	4,1	4,5
6	2	16	107,2	5,6	7,4	103,3	4,6	5,5
6	3	16	190,3	3,1	6,3	188,4	4,4	5,1
6	4	16	11,4	11,1	11,1	11,5	8,0	13,5
6	5	16	136,7	4,5	4,5	140,4	3,9	5,5
6	6	16	276,6	1,5	5,7	267,3	1,3	3,1
6	7	16	36,5	5,1	12,4	35,4	6,4	12,5
6	8	16	2054,8	2,1	5,0	1996,8	3,4	4,0
6	9	16	3697,5	3,6	7,6	3733,9	2,4	6,8

Table S1. Repeatability and intermediate precision by sample for each lab. A model one-way-ANOVA with run as a random factor was used to calculate the repeatability %CV (within-assay variability) and Intermediate Precision %CV (between-assay variability) by laboratory.
