# Supplementary material for: Validation of a Harmonized Enzyme-Linked-Lectin-Assay (ELLA-NI) Based Neuraminidase Inhibition Assay Standard Operating Procedure (SOP) for Quantification of N1 Influenza Antibodies and the Use of a Calibrator to Improve the Reproducibility of the ELLA-NI With Reverse Genetics Viral and Recombinant Neuraminidase Antigens: A FLUCOP Collaborative Study
Source: Front Immunol. 2022 Jun 17;13:909297. doi: 10.3389/fimmu.2022.909297 (PMC9248865; doi:10.3389/fimmu.2022.909297)
Supplement: Supplementary file 8 [file Table_2.pdf]

Lab	N samples	N results used	IP CV (%) Singlicate Series 1	IP CV (%) Singlicate Series 2	IP CV (%) Intraplate duplicate	IP CV (%) Interplate duplicate
1	9	144	31,6	34,4	31,5	30,9
2	8	128	24,7	27,8	25,6	16,8
3	9	144	20,3	15,5	16,7	14,5
4	9	144	34,4	34,6	31,4	30,7
5	9	72	19,6	16,1	16,5	16,2
6	9	144	7,7	7,6	6,7	6,6

Table S2. Overall Intermediate Precision (IP) %CV for singleton and duplicate testing of samples. A mixed procedure of SAS and two-way-ANOVA, with the sample and the run as random factors, was performed on $\log_{10}$-transformed titres in order to calculate the overall precision for singleton and duplicate testing of sera samples. IP was calculated for each lab with results calculated from a single measurement (Singlicate series 1 or Singlicate series 2), results calculated with the two measurements on the same plate (Intraplate duplicate) or results calculated with the two measurements of the two series of plates (Interplate duplicate).
