# Supplementary material for: Validation of a Harmonized Enzyme-Linked-Lectin-Assay (ELLA-NI) Based Neuraminidase Inhibition Assay Standard Operating Procedure (SOP) for Quantification of N1 Influenza Antibodies and the Use of a Calibrator to Improve the Reproducibility of the ELLA-NI With Reverse Genetics Viral and Recombinant Neuraminidase Antigens: A FLUCOP Collaborative Study
Source: Front Immunol. 2022 Jun 17;13:909297. doi: 10.3389/fimmu.2022.909297 (PMC9248865; doi:10.3389/fimmu.2022.909297)
Supplement: Supplementary file 9 [file Table_3.pdf]

				Series 1		Series 2	
**Lab**	**Operator**	**N samples**	**N results used**	**Rep CV (%)**	**IP CV (%)**	**Rep CV (%)**	**IP CV (%)**
1	1	9	72	26,5	26,5	31,4	32,4
1	2	9	72	29,9	30,0	20,6	21,0
2	1	8	64	14,8	18,1	19,7	19,9
2	2	8	64	24,2	24,2	26,3	31,9
3	1	9	72	17,4	18,1	13,2	13,5
3	2	9	72	14,7	16,1	11,9	13,6
4	1	9	72	34,7	36,8	33,8	35,4
4	2	9	72	28,8	28,8	29,7	31,1
5	1	9	72	17,6	18,2	15,2	15,2
6	1	9	72	7,8	8,0	7,3	7,9
6	2	9	72	5,9	6,6	6,7	6,7

Table S3. Repeatability and Intermediate precision (IP) 0CV by operator. A model 2-way-ANOVA with run and sample and their interaction as random qualitative factors was used to calculate precision by operator. Repeatability %CV (within-assay variability) and Intermediate Precision (IP) %CV (between-assay variability) are shown per lab, per operator.
